# Clinical pharmacokinetic-pharmacodynamic relationships between drug exposure and the QT/QTc interval: Protocol for a systematic review and quantitative synthesis

**DOI:** 10.1371/journal.pone.0357131

**Published:** 2026-10-28

**Authors:** Hoornaz Molana, Hadi Esmaily, Ghader Mohammadnezhad

**Affiliations:** 1 School of Medicine, Shahid Beheshti University of Medical Sciences, Tehran, Iran; 2 Department of Clinical Pharmacy, School of Pharmacy, Shahid Beheshti University of Medical Sciences, Tehran, Iran; 3 Department of Clinical Pharmacy, School of Pharmacy, Shahid Beheshti University of Medical Sciences, Tehran, Iran; Makerere University, UGANDA

## Abstract

Drug-induced QT/QTc prolongation is a central component of cardiac safety assessment. Concentration-QT/QTc modelling uses measured or model-predicted systemic drug exposure to estimate the magnitude and uncertainty of effects on ventricular repolarisation and can support regulatory decision-making in early clinical development. However, the evidence is dispersed across dedicated thorough QT studies, early-phase studies, population pharmacokinetic-pharmacodynamic analyses, and secondary pooled analyses, with substantial variation in exposure metrics, electrocardiographic acquisition, heart-rate correction, model structure, assessment of active metabolites and delayed effects, and reporting of uncertainty. This protocol describes a systematic review of quantitative human concentration-QT/QTc evidence. PubMed/MEDLINE, Embase, Scopus, the Cochrane Central Register of Controlled Trials, ClinicalTrials.gov, and Google Scholar will be searched from inception without date or language restrictions. Original human studies will be eligible when drug or metabolite concentrations are measured or estimated from a pharmacokinetic model informed by study data and are quantitatively related to QT or QTc outcomes. Two reviewers will independently screen records, extract data, and assess methodological limitations. The primary estimands will be the concentration-QT/QTc drug-effect parameter and the predicted placebo-adjusted change from baseline in QTc at a clinically relevant exposure. Random-effects meta-analysis will be undertaken only within the same active moiety when analyte, endpoint, correction method, model estimand, and exposure scenario are sufficiently comparable; otherwise, structured synthesis without meta-analysis will be performed. Risk of bias will be assessed using design-appropriate tools where applicable, supplemented by a prespecified concentration-QT/QTc methodological framework. Certainty will be evaluated for coherent drug-specific bodies of evidence. This review will provide a transparent map of clinical concentration-QT/QTc methods and estimates and identify priorities for future cardiac safety studies. **PROSPERO registration**: CRD420261469142.

## Introduction

Drug-induced prolongation of the QT interval reflects delayed ventricular repolarisation and is used as a biomarker of proarrhythmic liability during clinical drug development. Although QT prolongation is an imperfect surrogate for torsades de pointes (TdP), clinically relevant changes in heart-rate-corrected QT (QTc) remain an important component of the totality of evidence used to assess cardiac safety and sudden cardiac death (SCD) risk assessment. The International Council for Harmonisation E14/S7B questions and answers recognise concentration-response analysis as a primary basis for characterising a drug’s QTc effect when the clinical study and electrocardiographic assessment are adequately designed [1].

Concentration-QT/QTc analysis differs from a simple comparison of administered doses or scheduled time points. It uses observed or model-predicted concentrations to characterise the exposure-response relationship and to estimate the mean QTc effect, with uncertainty, at a concentration of regulatory or clinical interest. The scientific white paper by Garnett and colleagues established a commonly used linear mixed-effects framework incorporating centred baseline QTc, nominal time, treatment, and concentration [2]. Prospective work from the IQ-Cardiac Safety Research Consortium demonstrated that robust electrocardiographic sampling and exposure-response analysis in early-phase studies can detect small QTc effects and, in appropriate circumstances, replace a dedicated thorough QT study [3,4].

The apparent simplicity of a concentration-QTc slope can obscure important methodological variation. Studies differ in the populations enrolled, exposure range achieved, bioanalytical sampling, temporal matching of pharmacokinetic and electrocardiographic measurements, use of replicate electrocardiograms, heart-rate correction, baseline and placebo adjustment, treatment of circadian effects, active metabolite assessment, and model diagnostics. A direct linear model assumes both linearity and absence of a delay between concentration and effect. These assumptions may be inappropriate when the exposure-response relationship is nonlinear, when a metabolite contributes to the effect, or when hysteresis is present. Consequently, estimates reported as concentration-QT/QTc relationships may not represent the same estimand and may not be directly comparable [5–8].

Previous publications have primarily provided methodological guidance, narrative reviews, regulatory perspectives, or practical tutorials on the design, analysis, and interpretation of concentration–QT/QTc studies. These works have established recommended modelling approaches and described the evolving role of concentration–QT/QTc analysis in replacing or complementing dedicated thorough QT studies. However, to our knowledge, no systematic review has comprehensively identified the original human clinical evidence across medicinal products while simultaneously extracting drug-specific quantitative concentration–QT/QTc estimates, characterising heterogeneity in exposure metrics and model structures, evaluating ECG–PK alignment and exposure coverage, assessing the treatment of active metabolites and delayed effects, and determining when estimates are sufficiently comparable for quantitative synthesis. The present review is designed to address this evidence-synthesis gap rather than to provide another methodological tutorial on how concentration–QT/QTc modelling should be performed [9,10].

A rigorous synthesis requires more than pooling all reported slopes. Concentration units, therapeutic exposure ranges, analytes, model structures, and QT correction methods differ substantially between drugs. Therefore, this review will prioritise drug-specific and estimand-specific synthesis, distinguish direct concentration models from analyses based on summary exposure metrics, and avoid quantitatively combining non-comparable effects. The review will also develop and apply a transparent concentration-QT/QTc-specific methodological appraisal alongside conventional risk-of-bias tools.

The objective of this protocol is to prespecify the eligibility criteria, information sources, data items, appraisal methods, and statistical synthesis plan for a systematic review of clinical concentration-QT/QTc analyses of medicinal products in humans.

## Review objectives

The primary objective is to systematically identify, critically appraise, and synthesise human studies that quantitatively evaluate the relationship between systemic concentrations of medicinal products or their metabolites and QT/QTc outcomes.

- Which medicinal products and drug classes have been evaluated using concentration-QT/QTc or exposure-QT/QTc analyses in humans?
- Which study designs, populations, exposure measures, electrocardiographic acquisition methods, QT correction formulas, and pharmacokinetic-pharmacodynamic model structures have been used?
- What are the direction, magnitude, and uncertainty of reported concentration-QT/QTc drug-effect parameters and predicted QT/QTc effects at clinically relevant exposures?
- How do reported estimates vary according to population, exposure range, analyte, QT correction method, study design, model structure, active metabolite assessment, and evaluation of nonlinear or delayed effects?
- Which methodological and reporting limitations affect the interpretation, comparability, and generalisability of existing analyses?
- Where data are sufficiently comparable, what are the pooled drug-specific concentration-QT/QTc estimates and their between-study heterogeneity?

## Materials and methods

### • Protocol design and reporting

As reported in S1 File, this protocol was developed in accordance with the Preferred Reporting Items for Systematic Review and Meta-Analysis Protocols (PRISMA-P) 2015 statement and explanation and elaboration documents [11,12]. The final review will be reported according to PRISMA 2020 [13]. If quantitative pooling is inappropriate, the structured synthesis will follow the Synthesis Without Meta-analysis (SWiM) instructions [14]. Fig 1 summarises the framework linking eligible clinical concentration-QT/QTc studies to planned drug-specific evidence synthesis.

**Fig 1:**
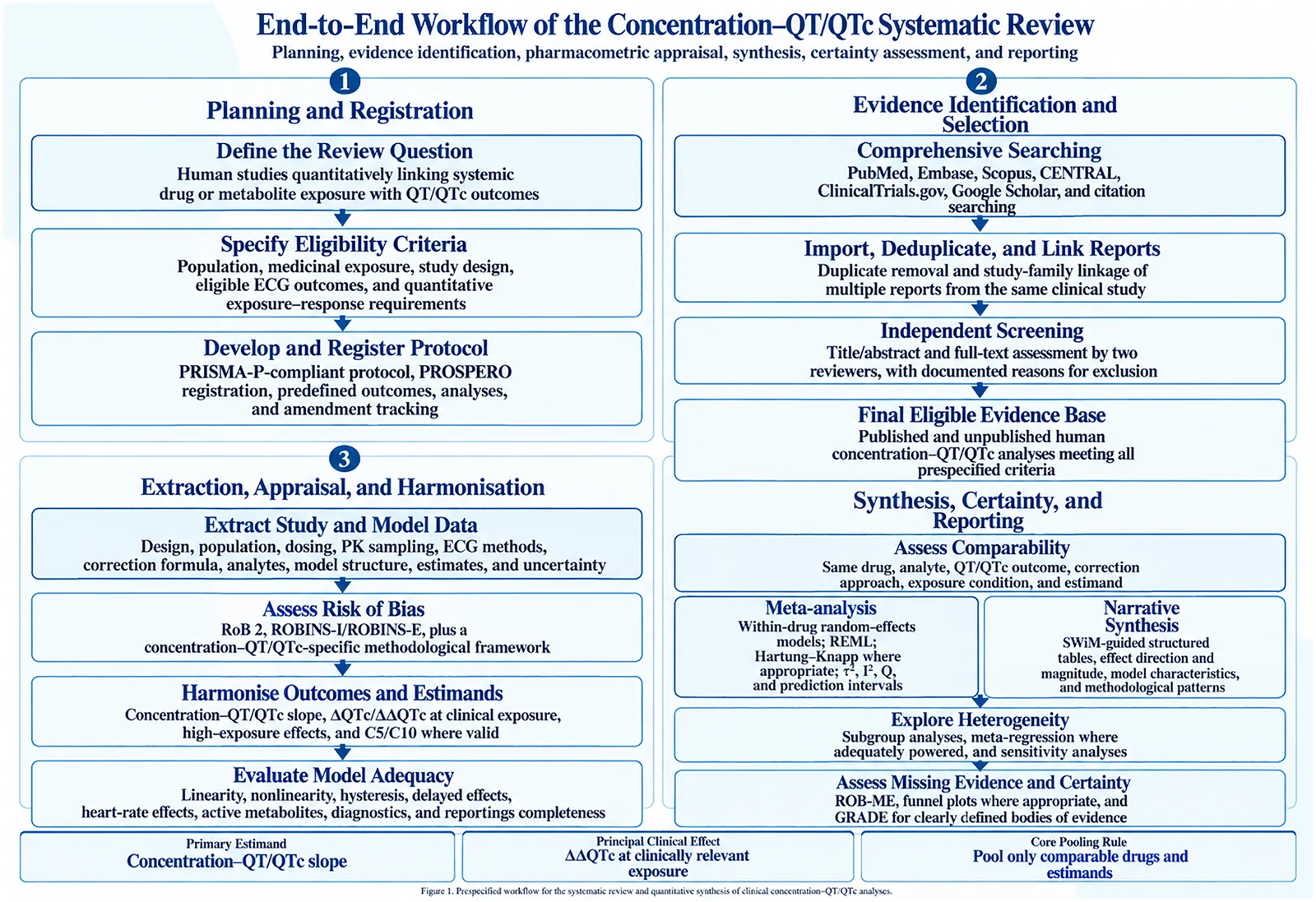
Conceptual framework linking eligible clinical concentration-QT/QTc studies to planned drug-specific evidence synthesis. PK, pharmacokinetic; ECG, electrocardiogram; ΔΔQTc, placebo-adjusted change from baseline in corrected QT; SWiM, Synthesis Without Meta-analysis.

### • Registration and protocol amendments

The review was prospectively registered in the International Prospective Register of Systematic Reviews (PROSPERO; CRD420261469142). Any amendment made after protocol registration will be documented in a dated amendment log describing the original method, revised method, reason for the change, and stage of the review at which the change occurred. Amendments affecting eligibility, outcomes, risk-of-bias (RoB) assessment, or synthesis will be reported in both the PROSPERO record and the final review.

### • Review status and timeline

The systematic review is currently ongoing. The literature search has been initiated, and record screening is currently in progress. At the time of submission of this protocol manuscript, data extraction, risk-of-bias assessment, quantitative synthesis, and interpretation of the review findings have not been completed, and no review results have been generated.

Title/abstract and full-text screening are expected to be completed by September 2026. Data extraction and methodological appraisal are expected to be completed by October 2026. Quantitative and structured evidence synthesis is expected to be completed thereafter, with the results of the systematic review anticipated by November 2026. Because this is a systematic review, no a priori sample-size calculation is applicable; all eligible studies identified through the prespecified search and screening process will be included.

### • Eligibility criteria

Eligibility will be defined using a population-exposure-outcome-study design framework. Table 1 summarises the eligibility criteria.

**Table 1:** Eligibility criteria for the systematic review.

Domain	Inclusion criteria	Exclusion criteria
**Population**	Human participants of any age, sex, ethnicity, or clinical condition, including healthy volunteers and patients. Studies in special populations, including renal or hepatic impairment and drug-drug interaction settings, are eligible.	Animal, in vitro, ex vivo, isolated-tissue, cellular, or purely simulated studies. Mixed human/non-human reports are eligible only when human data are separable.
**Exposure**	Any medicinal product administered by any route, dose, or regimen. Parent drug and relevant metabolites are eligible. Concentrations may be observed or individually predicted from a pharmacokinetic model informed by measured study data.	Environmental chemicals, non-medicinal exposures, illicit substances, acute poisoning, and overdose studies. Purely hypothetical or simulation-only exposure profiles are excluded.
**Exposure-response analysis**	A formal quantitative model or analysis relating a continuous systemic concentration or exposure metric to QT/QTc. Direct time-matched concentration models are primary. C_max_-, C_min_-, or AUC-based analyses are eligible only when the exposure metric is a continuous predictor and will be synthesised separately.	Dose-only, treatment-assignment-only, or duration-only analyses; therapeutic drug monitoring reports without a QT/QTc model; pharmacokinetic studies without a QT/QTc component.
**Outcome**	QT, QTcF, QTcB, individually corrected QTc, study-specific QTc, ΔQTc, or ΔΔQTc* assessed by standard 12-lead ECG, validated digital ECG, or validated continuous ECG/Holter methodology.	Categorical ECG abnormalities or QT prolongation without a quantitative exposure-response analysis.
**Study design**	Original randomised or non-randomised human studies, crossover or parallel studies, thorough QT studies, single- or multiple-ascending-dose studies, phase I-IV studies, drug-drug interaction and organ-impairment studies, observational PK/PD studies, and published pooled or secondary analyses of individual-level data.	Reviews, meta-analyses, protocols, editorials, commentaries, letters without original data, purely methodological papers without original human data, and descriptive case reports/series without a formal prespecified exposure-response model.
**Publication status and language**	Full articles, detailed regulatory or technical reports identified through eligible records, theses, and conference reports with sufficient quantitative information. No date or language restriction at the search stage.	Records for which eligibility and results remain unassessable after reasonable attempts to obtain or translate the report will be listed as awaiting classification rather than silently excluded.

AUC: Area Under the Curve, C_max_: Maximum or Peak Concentration, C_min_: Minimum or Nadir Concentration, ECG: Electrocardiogram, PK/PD: Pharmacokinetic/pharmacodynamic.

* **QT**: Time from QRS onset to T-wave end; **QTc**: QT interval corrected for heart rate; **QTcF**: Fridericia-corrected QT; **QTcB**: Bazett-corrected QT; **QTcI**: Individually corrected QT; **QTcS**: Study-specific QTc; **ΔQTc**: Post-dose change in QTc from baseline; **ΔΔQTc**: Placebo-adjusted QTc change from baseline.

A study will be eligible if, for example, individual time-matched plasma concentrations are related quantitatively to ΔQTcF using a linear or nonlinear concentration–effect model. Studies relating a continuous summary exposure metric, such as individual Cmax, Cmin, or AUC, to QT/QTc will also be eligible, but these analyses will be classified separately from direct time-matched concentration–QT/QTc models. Analyses using individual concentrations predicted from a pharmacokinetic or population pharmacokinetic model will be eligible when that model is informed by measured human pharmacokinetic data from the study or a justified linked clinical dataset. In contrast, comparisons of QT/QTc across administered dose groups without a quantitative exposure–response analysis will not be eligible. Analyses comparing only categorical exposure groups, such as low-, medium-, and high-Cmax categories, without modelling exposure as a continuous predictor will also be excluded. Purely simulation-based analyses without original human concentration–QT/QTc data, and studies reporting QT/QTc outcomes without a quantitative relationship to systemic exposure, will not meet the eligibility criteria.

### • Population and setting

Studies conducted in any clinical or research setting will be eligible, including dedicated phase I units, clinical pharmacotherapy centres, hospitals, outpatient clinics, specialist treatment centres, and community-based settings. No restrictions will be applied by country, geographic region, healthcare system, level of care, baseline QT risk, or underlying clinical condition.

### • Exposure definition

The primary exposure of interest is a measured or model-predicted systemic concentration of the parent drug or a relevant metabolite temporally linked to a QT/QTc observation. Model-predicted concentrations will be eligible only when derived from a pharmacokinetic model informed by measured concentrations from the study population or a clearly justified external population model applied to the study participants. Summary exposure metrics such as C_max_ or AUC will be included only if they are evaluated as continuous predictors of QT/QTc; these estimates will not be combined with direct time-matched concentration-QT/QTc slopes.

### • Comparator

Placebo-controlled, active-controlled, baseline-adjusted, and non-comparative studies will be eligible. Where available, placebo-adjusted change from baseline (ΔΔQTc) will be prioritised because placebo adjustment separates drug-related effects from time-dependent and baseline variation.

### • Information sources

The following sources will be searched from inception to the final search date: PubMed/MEDLINE, Embase, Scopus, the Cochrane Central Register of Controlled Trials, ClinicalTrials.gov, and Google Scholar. Both published and unpublished or incompletely published studies will be sought. Reference lists of included studies and relevant methodological or clinical reviews will be screened, and forward citation searching will be performed in Scopus and Google Scholar. Trial registry records, supplementary appendices, and regulatory or technical reports linked to potentially eligible studies will be examined for complementary data. Searches will be updated immediately before the final synthesis.

### • Search strategy

The search strategy combines terms for QT/QTc or ventricular repolarisation with terms for concentration, systemic exposure, pharmacokinetics, pharmacokinetic-pharmacodynamic modelling, exposure-response analysis, and thorough QT studies. Controlled vocabulary and free-text terms will be adapted to each database. No publication-date or language limit will be applied. The search syntaxes for each database are provided in S2 File.

### • Google Scholar and trial registry searches

Because Google Scholar and ClinicalTrials.gov do not provide the same reproducible field and proximity functions as bibliographic databases, multiple shorter searches will be run separately. Exact phrases will include “concentration-QT,” “concentration-QTc,” “exposure-QT,” “exposure-QTc,” “thorough QT,” “PK-PD QTc,” “hysteresis QTc,” and “effect compartment QTc.” For Google Scholar, the first 200 records ranked by relevance for each prespecified query will be screened, with the date, exact query, total displayed results, and number screened recorded. ClinicalTrials.gov will be searched without restrictions on recruitment status, phase, age, country, or completion status.

### • Data management

Records will be exported to a reference manager and deduplicated using automated matching followed by manual verification. Screening decisions, exclusion reasons, and study-family links will be maintained in a systematic-review platform or a version-controlled database. Each underlying clinical study will receive a unique study identifier, and multiple publications, registry entries, appendices, and regulatory reports arising from the same study will be linked. Data extraction forms and decision rules are shown in S3 File.

### • Study selection

Two reviewers will independently screen titles and abstracts against the eligibility criteria. Potentially eligible records will undergo independent full-text assessment by the same reviewers. Disagreements will be resolved by discussion; unresolved disagreements will be adjudicated by a third reviewer. Reasons for full-text exclusion will be recorded using mutually exclusive categories. When a report contains multiple analyses, eligibility will be determined at the level of the specific concentration-QT/QTc result. The final review will include a PRISMA 2020 flow diagram. A pilot exercise will be performed on a sample of records before formal screening to refine operational definitions without changing the registered scope.

### • Data collection process

A detailed extraction form will be developed and pilot tested on at least five diverse studies, including a thorough QT study, an early-phase concentration-QTc study, and a nonlinear or parent-metabolite analysis where available. Two reviewers will independently extract study characteristics and all primary quantitative results. Discrepancies will be resolved by consensus or third-reviewer adjudication. Where relevant information is unclear or missing, corresponding authors will be contacted by email on up to two occasions at least two weeks apart. Individual participant data will not be sought; requests will be limited to missing aggregate estimates, model parameters, variance measures, and methodological clarification. Values reported only in figures may be digitised using validated software, and such derivations will be flagged and tested in sensitivity analyses. When a study reports multiple eligible concentration–QT/QTc models or QT correction methods, both reviewers will initially extract all eligible analyses and independently apply the prespecified outcome hierarchy. For QT correction, placebo-adjusted QTcF will be prioritised when available, followed by individually corrected QTc, the study-prespecified primary correction, QTcB, and uncorrected QT. For model selection, the prespecified primary model will be prioritised; if no primary model is explicitly identified, the model designated as primary by the investigators and supported by the reported diagnostics will be selected. Alternative and exploratory models will remain recorded as secondary estimates. Disagreement regarding model classification, QT correction priority, or selection of the estimate for synthesis will first be resolved by discussion and, if unresolved, by a third reviewer. All adjudication decisions affecting the synthesis dataset will be documented in a decision log.

### • Data items

The prespecified data domains are presented in Table 2. Data will be extracted separately for each eligible concentration-QT/QTc model and target exposure within a study. A detailed data extraction form for each domain is shown in S3 File.

**Table 2:** Prespecified data-extraction domains.

Domain	Variables to be extracted
**Study identification**	Authors, year, country, sponsor/funding, registry identifier, study identifier, publication type, related reports.
**Design and population**	Phase, randomisation, blinding, crossover/parallel/single-arm design, control groups, sample size analysed, age, sex, health status, indication, comorbidities, organ impairment, baseline QT risk.
**Drug and dosing**	Drug, formulation, route, dose levels, single/multiple dose, duration, steady state, supratherapeutic strategy, drug-drug interaction, food condition.
**Pharmacokinetics**	Analyte, parent/metabolite, bioanalytical matrix and assay, sampling schedule, observed or model-predicted concentration, exposure range, C_max_/C_min_/AUC, below-quantification handling, population-PK model source.
**ECG**	ECG modality, extraction method, central/local reading, reader blinding, replicate ECGs, timing relative to PK sample, QT and RR measurement, heart-rate correction, baseline definition, heart-rate effect.
**Model**	Outcome scale, direct/indirect model, linear/log-linear/E_max_/sigmoid E_max_/effect-compartment/joint parent-metabolite model, fixed and random effects, nominal-time terms, baseline centring, covariates, model-selection procedure, software.
**Quantitative results**	Slope or drug-effect parameter with unit and uncertainty; predicted ΔQTc/ΔΔQTc at therapeutic and high clinical exposure; concentration associated with 5- or 10-ms effect; intercept; regulatory threshold conclusion; subgroup estimates.
**Diagnostics and robustness**	Goodness-of-fit, residual diagnostics, visual predictive checks, linearity assessment, hysteresis assessment, sensitivity analyses, missing-data handling, outlier rules, uncertainty method.
**Reporting and transparency**	Protocol or analysis-plan availability, prespecification, author contact, conflicts of interest, data/code availability, selective reporting concerns.

### • Outcomes and prioritisation

Two primary estimands will be sought. First, the drug-effect parameter from the prespecified concentration-QT/QTc model, most commonly a slope expressed as milliseconds (ms) per concentration unit (e.g., ms/mg/L), together with its standard error (SE), confidence interval (CI), credible interval, or variance. For nonlinear models, the relevant model parameters and uncertainty will be extracted without forcing conversion to a linear slope. Second, the model-predicted placebo-adjusted change from baseline in QTc at a prespecified clinically relevant concentration will be extracted. The preferred clinically relevant concentration will be the geometric mean C_max_ at the highest approved or intended therapeutic regimen, or the study-defined high clinical exposure when justified by maximum dosing, accumulation, organ impairment, or a clinically relevant drug-drug interaction (DDI).

Secondary outcomes will include predicted QT/QTc change at supratherapeutic or worst-case clinical exposure; concentration associated with a 5-ms or 10-ms QTc increase; upper bound of the two-sided 90% CI at the target concentration; categorical ability to exclude an effect exceeding 10 ms; evidence of nonlinearity or delayed effect; contribution of active metabolites; effect on heart rate; and frequencies of model structures, correction methods, electrocardiographic procedures, and reporting practices. Concentrations associated with a 5-ms or 10-ms effect will be derived only when the reported model supports the calculation, the required parameters and covariance are available, and the target lies within or close to the observed exposure range. Extrapolations beyond the supported concentration range will not be performed.

When multiple QT correction methods are reported, placebo-adjusted change from baseline in Fridericia-corrected QT (ΔΔQTc) will be prioritised, followed by individually corrected QTc (QTcI), the study-prespecified primary correction (QTcS), Bazett-corrected QTc (QTcB), and uncorrected QT. Alternative corrections will be retained for sensitivity or subgroup analyses. When several models are reported, the prespecified primary model will be selected; if none is identified, the model judged primary by the investigators and supported by diagnostics will be used. Exploratory models will be extracted separately and will not replace a prespecified primary result without justification. Table 3 shows the outcome synthesis and reporting hierarchy.

**Table 3:** Outcome hierarchy and synthesis rules.

Priority	Estimand	Preferred form	Planned synthesis
**Primary (1)**	Concentration-QT/QTc drug-effect parameter	Slope with SE/CI for direct linear models; native parameters for nonlinear or delayed-effect models	Within-drug pooling only when analyte, endpoint, correction, and model estimand are comparable.
**Primary (2)**	Predicted QTc effect at clinically relevant exposure	Mean ΔΔQTcF in milliseconds with two-sided 90% CI at a clearly defined target concentration	Within-drug random-effects synthesis when target exposure definitions are comparable
**Secondary (1)**	Effect at high clinical or supratherapeutic exposure	ΔΔQTc in milliseconds with uncertainty	Separate from therapeutic-exposure synthesis
**Secondary (2)**	Concentration associated with 5- or 10-ms QTc increase	Reported or validly derived concentration with uncertainty	Narrative or drug-specific synthesis; no extrapolation beyond supported range
**Descriptive**	Methods and reporting	Model type, ECG/PK matching, correction, metabolite/hysteresis assessment, diagnostics	Structured tabulation and SWiM synthesis

### • RoB and methodological appraisal

Because the primary estimand is a model-based exposure-response result rather than necessarily a conventional between-group treatment effect, no single generic RoB tool is appropriate for every included study. Appraisal will therefore be layered. A concentration-QT/QTc-specific methodological framework will be applied to every eligible result. In addition, design-based tools will be applied when the target result includes an estimand for which the tool was developed: RoB 2, including the crossover version, for randomised comparisons [15]; ROBINS-I for non-randomised intervention comparisons [16]; and ROBINS-E for compatible observational cohort analyses of an exposure effect [17]. These tools will not be mechanically applied to single-arm pharmacometric models when their causal estimand is not represented by the tool.

The supplementary concentration-QT/QTc framework is informed by ICH E14/S7B and the scientific white paper [1,2]. It will assess domains not fully captured by generic tools, as shown in Table 4. Judgements will be low risk, some concerns, high risk, or insufficient information. No numerical summary score will be calculated. An overall methodological judgement will consider the importance and likely direction of bias rather than an arithmetic count of domains. The concentration–QT/QTc-specific methodological appraisal framework was developed specifically for this systematic review and has not previously been validated or applied as a standalone RoB instrument. It is intended to supplement, rather than replace, established design-based RoB tools by addressing concentration–QT/QTc-specific methodological issues that are not fully captured by generic instruments. Its domains were informed by ICH E14/S7B recommendations, the concentration–QTc scientific white paper, and established pharmacometric principles. Before formal appraisal, two reviewers will independently apply the framework to a calibration sample of methodologically diverse eligible studies, including, where available, a conventional linear concentration–QTc analysis, a thorough QT study, and a nonlinear or parent–metabolite analysis. Differences in interpretation will be discussed and the operational guidance refined before application to the full evidence base, without changing the predefined domains on the basis of study results. Initial domain-level judgments will remain independent until inter-reviewer reliability has been assessed. Agreement will be reported descriptively using percentage agreement and, when the number of assessments permits meaningful estimation, Cohen’s κ with 95% confidence intervals. Final disagreements will be resolved by consensus or third-reviewer adjudication.

**Table 4:** Concentration-QT/QTc-specific methodological appraisal domains.

Domain	Key signalling considerations
**Study design and population**	Does the design support the stated estimand? Are participant selection, withdrawals, and analysis population adequately described?
**Exposure range**	Were therapeutic and clinically relevant high exposures adequately represented without unsupported extrapolation?
**PK measurement and prediction**	Was the bioanalytical method validated? Were sampling times adequate? Were predicted concentrations based on a justified model?
**ECG acquisition and reading**	Were ECG methods validated, time matched, replicated, centrally read or blinded where appropriate, and adequately described?
**Heart-rate correction and time effects**	Was the correction method appropriate? Were drug-related heart-rate changes, baseline, placebo, and nominal-time/circadian effects addressed?
**Parent drug and metabolites**	Were QT-active metabolites considered or was their omission justified? Was joint modelling appropriate when both contributed?
**Model specification**	Was the primary model prespecified? Were fixed/random effects and covariates justified? Was concentration treated on an appropriate scale?
**Linearity and delayed effects**	Were nonlinearity and hysteresis assessed when suggested by data or pharmacology? Were alternative models compared transparently?
**Missingness, BLQ data, and outliers**	Were missing ECG/PK observations, below-quantification concentrations, protocol deviations, and outliers handled appropriately?
**Diagnostics and uncertainty**	Were goodness-of-fit, residual diagnostics, predictive checks, parameter covariance, and confidence intervals adequate?
**Selective analysis reporting**	Were multiple models or endpoints selectively reported? Are protocols, analysis plans, registries, or related reports consistent with the publication?

BLQ: Below the lower limit of quantification, ECG: Electrocardiogram, PK: Pharmacokinetic

### • Data synthesis

#### a. Descriptive and structured synthesis.

All included studies will be presented in structured tables by active moiety. Study and population characteristics, exposure conditions, ECG methods, correction formulas, analytes, model structures, diagnostics, and quantitative results will be described. Direct time-matched concentration models will be separated from analyses using summary exposure metrics such as Cmax or AUC. Linear, nonlinear, delayed-effect, and joint parent-metabolite models will be grouped separately. Where meta-analysis is not appropriate, studies will be grouped by active moiety and estimand, effect estimates will be displayed with their precision and exposure range, and the direction and magnitude of findings will be summarised according to SWiM principles [14]. When direct quantitative pooling is not appropriate, model results will be retained in their native parameterisation rather than transformed artificially to a linear slope. Linear direct-effect models will be summarised using slopes and their uncertainty. For E_max_ models, E_max_ and EC50 will be extracted; for sigmoid E_max_ models, E_max_, EC_50_, and the Hill coefficient will be retained; for effect-compartment or other delayed-effect models, the reported effect-compartment or delay parameter together with the concentration–effect parameters will be summarised; and for joint parent–metabolite models, parent- and metabolite-specific parameters or the reported joint prediction will be presented as appropriate. Model-specific estimates will be tabulated together with their uncertainty, exposure range, target exposure, and principal diagnostic findings. Models with structurally different estimands will not be converted to a common slope solely for meta-analysis. Where several studies of the same active moiety use comparable model structures, parameters may be summarised within that model class. Otherwise, findings will be synthesised according to SWiM principles. Model-predicted QT/QTc effects at clinically relevant exposures may additionally provide a common clinically interpretable summary when these predictions are defined comparably and are supported by the observed exposure range.

#### b. Criteria for quantitative synthesis.

Quantitative synthesis will be considered only when at least three independent studies evaluate the same active moiety and provide a sufficiently comparable analyte, QT/QTc endpoint, heart-rate correction, exposure-response estimand, target exposure, and model structure. Different salts or formulations of the same active moiety may be combined only when systemic analyte exposure is directly comparable. Parent-drug and metabolite estimates will be synthesised separately unless a joint model produces a single clinically interpretable prediction. Raw slopes will not be pooled across different active moieties because concentration scales, molecular potency, therapeutic exposure ranges, and active metabolites differ.

### • Effect measures and harmonisation

For linear direct-effect models, the effect measure will be the change in QT/QTc in ms per common concentration unit. Within an active moiety, mass concentration units will be converted to a prespecified unit, preferentially ng/mL or µg/mL as appropriate, with all conversion factors independently verified. Molar conversion will be used only for exploratory presentation and not to justify cross-drug pooling. For predicted effects, the effect measure will be the mean QT/QTc or ΔΔQTc change in milliseconds at a prespecified concentration. Standard errors will be extracted directly or derived from valid confidence intervals or credible intervals. A two-sided 90% CI will be retained where reported for regulatory concentration-QTc estimates; if synthesis requires a sampling variance, it will be converted using the corresponding normal quantile.

### • Meta-analysis model

Random-effects meta-analysis using restricted maximum likelihood estimation will be the default because genuine between-study heterogeneity is expected. Hartung-Knapp adjustment will be used for interval estimation, with conventional Wald intervals reported in sensitivity analyses when the number of studies is small, or the adjustment behaves anomalously [18]. The pooled estimate, 95% confidence interval, between-study variance (τ²), I², Cochran Q, and a 95% prediction interval will be reported when estimable. τ^2^ and prediction intervals will be emphasised because I^2^ may be unstable when few studies are available. Analyses will be performed in R using the metafor package or equivalent validated software [19].

### • Dependent estimates and multiple reports

When multiple eligible estimates arise from the same underlying study, dependence will be addressed by a prespecified hierarchy or, when sufficient data are available, multilevel or multivariate meta-analysis. The primary hierarchy will select: the prespecified primary model; placebo-adjusted change from baseline; QTcF or individually corrected QTc; the clinically relevant target exposure; and the analysis population defined as primary by the study. Separate dose cohorts contributing to a single concentration-QT/QTc model will not be treated as independent studies. Multiple publications from the same study will be combined into one study record, with complementary data extracted from each report.

### • Exploration of heterogeneity

Potential sources of heterogeneity include study design, healthy-volunteer versus patient population, age group, exposure range, single- versus multiple-dose administration, placebo adjustment, QT correction method, ECG replication and reading method, parent versus metabolite analyte, model structure, prespecification, and assessment of nonlinearity or hysteresis. Subgroup analyses will be conducted only when categories contain adequate data. Meta-regression will be considered when at least ten independent studies contribute to a sufficiently comparable synthesis and, as a general rule, no more than one moderator will be fitted per ten studies. Analyses will be interpreted as exploratory and will not be used to infer causal explanations for heterogeneity.

### • Sensitivity analysis

Prespecified sensitivity analyses will exclude studies at high RoB or high methodological concern; conference abstracts without a full report; estimates derived from figures; studies using QTcB when a preferred correction is unavailable; non-prespecified or exploratory models; analyses requiring substantial variance reconstruction; and studies that do not assess relevant metabolites. Alternative analyses will compare restricted maximum likelihood with another τ^2^ estimator, Hartung-Knapp with conventional intervals, and primary versus alternative outcome hierarchies. Analyses based on summary exposure metrics will remain separate from direct concentration-QT/QTc models.

The principal analyses are the drug-specific synthesis of the prespecified primary concentration–QT/QTc model parameter and the predicted QT/QTc effect at the prespecified clinically relevant exposure. Prespecified sensitivity analyses described above will be considered supportive rather than primary analyses. Meta-regression analyses, subgroup analyses undertaken to explore observed heterogeneity, molar-scale presentations, analyses based on non-primary or exploratory models, analyses requiring substantial reconstruction of variance, and any additional analyses prompted by patterns identified after data extraction will be considered exploratory. Any post hoc exploratory analysis not specified in this protocol will be clearly identified as such in the final review and will not be interpreted as confirmatory evidence.

### • Assessment of reporting bias and missing evidence

Published reports will be compared with trial registry entries, protocols, statistical analysis plans, supplementary files, related publications, and regulatory or technical reports where available. Particular attention will be paid to selective omission of concentration-QT/QTc models, model specifications, exposure ranges, active metabolites, uncertainty estimates, and unfavourable or non-significant results. For sufficiently coherent meta-analyses, risk of bias due to missing evidence will be assessed using ROB-ME principles, recognising that the tool was developed primarily for intervention meta-analyses [20]. Funnel plots and regression-based tests for small-study effects will be considered only when at least ten independent studies contribute to a homogeneous synthesis.

### • Certainty of evidence

Grading of Recommendations, Assessment, Development and Evaluation (GRADE) will be applied to coherent drug-specific bodies of evidence for the principal clinically interpretable findings, including predicted ΔΔQTc at therapeutic or high clinical exposure and, where appropriate, pooled direct concentration-QT/QTc parameters [21]. Certainty will be classified as high, moderate, low, or very low after considering RoB, inconsistency, indirectness, imprecision, and publication bias. Randomised and non-randomised evidence will be assessed separately when they address materially different estimands. The presence of a concentration-response gradient will not automatically justify upgrading because an exposure-response analysis is an eligibility criterion and the subject of the review. Descriptive methodological findings, such as the frequency of model structures or reporting practices, will not receive a formal GRADE rating. Two reviewers will independently make GRADE judgements, with consensus or third-reviewer resolution.

### • Ethics and dissemination

Ethics approval and informed consent are not required because the review will use aggregate data from published or publicly accessible sources and will not obtain individual participant data. Findings will be disseminated through a peer-reviewed publication and scientific presentations. The final review, extraction form, aggregate dataset, amendment log, and complete search histories will be deposited in an open repository at or before publication, subject to copyright restrictions on source documents.

## Discussion

This protocol prespecifies a systematic approach to an evidence base that is clinically important but methodologically heterogeneous. Concentration-QT/QTc modelling has become central to contemporary cardiac safety assessment, yet published studies vary in the estimand reported and in the extent to which model assumptions, ECG quality, exposure coverage, metabolites, and uncertainty are documented. By distinguishing the drug-effect parameter from the predicted effect at a clinically relevant exposure, the review will retain both pharmacometric and clinical interpretability.

A major strength is the planned separation of non-comparable evidence. Raw slopes will not be pooled across different drugs, direct time-matched concentration models will not be combined with summary exposure-metric analyses, and nonlinear or delayed-effect models will not be forced into a linear framework. The use of study-family identifiers will also reduce double counting when several publications or regulatory reports describe the same clinical study.

The review also has anticipated limitations. Many concentration-QT/QTc analyses are secondary or regulatory analyses and may be incompletely reported in journal articles. Variance estimates and covariance matrices may be unavailable, limiting quantitative synthesis or derivation of predicted effects. Generic risk-of-bias tools were not designed specifically for pharmacometric exposure-response models; therefore, the supplementary methodological framework will be necessary but should not be represented as a validated standalone risk-of-bias instrument. The diversity of medicinal products means that many results will remain drug-specific and unsuitable for pooled analysis.

Despite these constraints, the planned review will provide a structured inventory of clinical concentration-QT/QTc evidence, identify recurring methodological weaknesses, and clarify which drug-specific estimates can be quantitatively synthesised. The findings may inform future study design, analysis planning, reporting standards, and interpretation of clinical cardiac safety data.

## Supporting information

S1 File PRISMA-P 2015 checklist. (DOCX)

S2 File Search syntaxes for each database. (PDF)

S3 File Draft data-extraction form and concentration–QT/QTc methodological appraisal form. (DOCX)
