# Supplementary material for: Clinical pharmacokinetic-pharmacodynamic relationships between drug exposure and the QT/QTc interval: Protocol for a systematic review and quantitative synthesis
Source: PLoS One. 2026 Oct 28;21(10):e0357131. doi: 10.1371/journal.pone.0357131 (PMC13716211; doi:10.1371/journal.pone.0357131)
Supplement: Supplementary file 1 [file pone.0357131.s002.pdf]

Search strategies

No restrictions will be applied according to publication date or language. Searches will be updated before the final analysis. Search dates and the number of records retrieved from each source will be documented when the searches are executed.

1. PubMed, including MEDLINE

Search interface

PubMed

Search strategy

```
(
 (
  "concentration QT"[tiab:~3]
  OR "concentration QTc"[tiab:~3]
  OR "exposure QT"[tiab:~3]
  OR "exposure QTc"[tiab:~3]
  OR "concentration corrected QT"[tiab:~5]
  OR "exposure corrected QT"[tiab:~5]
  OR "concentration response QT"[tiab:~5]
  OR "exposure response QT"[tiab:~5]
  OR "concentration dependent QT"[tiab:~4]
  OR "exposure dependent QT"[tiab:~4]
  OR "plasma concentration QT"[tiab:~5]
  OR "plasma concentration QTc"[tiab:~5]
  OR "serum concentration QT"[tiab:~5]
  OR "serum concentration QTc"[tiab:~5]
  OR "drug concentration QT"[tiab:~5]
  OR "drug concentration QTc"[tiab:~5]
 )

 OR

 (
  (
   QT[tiab]
   OR QTc[tiab]
   OR QTcF[tiab]
   OR QTcB[tiab]
   OR "QT interval"[tiab]
   OR "corrected QT"[tiab]
   OR "rate-corrected QT"[tiab]
   OR "heart rate corrected QT"[tiab]
   OR "QT prolongation"[tiab]
   OR "cardiac repolarization"[tiab]
```

```
  OR "ventricular repolarization"[tiab]
)

AND

(
  concentration*[tiab]
  OR exposure[tiab]
  OR exposures[tiab]
  OR "systemic exposure"[tiab]
  OR Cmax[tiab]
  OR Cmin[tiab]
  OR Css[tiab]
  OR "maximum concentration"[tiab]
  OR "peak concentration"[tiab]
  OR "trough concentration"[tiab]
  OR "steady-state concentration"[tiab]
  OR "steady state concentration"[tiab]
  OR "plasma concentration"[tiab]
  OR "plasma concentrations"[tiab]
  OR "serum concentration"[tiab]
  OR "serum concentrations"[tiab]
  OR "blood concentration"[tiab]
  OR "blood concentrations"[tiab]
  OR "drug concentration"[tiab]
  OR "drug concentrations"[tiab]
  OR "plasma level"[tiab]
  OR "plasma levels"[tiab]
  OR "serum level"[tiab]
  OR "serum levels"[tiab]
  OR metabolite*[tiab]
  OR pharmacokinetic*[tiab]
  OR pharmacodynamic*[tiab]
  OR PKPD[tiab]
  OR "PK PD"[tiab]
  OR "PK-PD"[tiab]
  OR "pharmacokinetic pharmacodynamic"[tiab]
  OR "pharmacokinetic-pharmacodynamic"[tiab]
  OR "Pharmacokinetics"[Mesh]
  OR "Drug Monitoring"[Mesh]
  OR
  (
    (
      AUC[tiab]
      OR "area under the curve"[tiab]
      OR "area under the concentration-time curve"[tiab]
```

```
    )
    AND
    (
      pharmacokinetic*[tiab]
      OR plasma[tiab]
      OR serum[tiab]
      OR drug[tiab]
    )
  )
)

AND

(
  model*[tiab]
  OR modelling[tiab]
  OR modeling[tiab]
  OR analys*[tiab]
  OR relationship*[tiab]
  OR association*[tiab]
  OR correlate*[tiab]
  OR correlation*[tiab]
  OR regression[tiab]
  OR slope[tiab]
  OR slopes[tiab]
  OR predict*[tiab]
  OR estimate*[tiab]
  OR "linear mixed effect"[tiab]
  OR "linear mixed effects"[tiab]
  OR "mixed effect model"[tiab]
  OR "mixed effects model"[tiab]
  OR "mixed-effect model"[tiab]
  OR "mixed-effects model"[tiab]
  OR pharmacometric*[tiab]
  OR Emax[tiab]
  OR "E-max"[tiab]
  OR nonlinear[tiab]
  OR "non-linear"[tiab]
  OR hysteresis[tiab]
  OR "effect compartment"[tiab]
  OR "effect-site compartment"[tiab]
  OR "exposure response"[tiab]
  OR "exposure-response"[tiab]
  OR "concentration response"[tiab]
  OR "concentration-response"[tiab]
  OR "Dose-Response Relationship, Drug"[Mesh]
```

```
      OR "Models, Statistical"[Mesh]
    )
  )

  OR

  (
    "thorough QT"[tiab]
    OR "thorough QTc"[tiab]
    OR "thorough QT/QTc"[tiab]
    OR
    (
      TQT[tiab]
      AND
      (
        QT[tiab]
        OR QTc[tiab]
        OR electrocardiogra*[tiab]
        OR cardiac[tiab]
      )
    )
  )
)

NOT
(
  Animals[Mesh]
  NOT Humans[Mesh]
)
```

2. Embase

Search interface

Embase.com

Combined search strategy

```
(
  (
    (
      concentration
      OR exposure
      OR 'systemic exposure'
      OR 'plasma concentration'
      OR 'serum concentration'
      OR 'blood concentration'
      OR 'drug concentration'
      OR 'drug level'
```

```
    OR 'plasma level'
    OR 'serum level'
  )
  NEAR/5
  (
    qt
    OR qtc
    OR qtcf
    OR qtcb
    OR 'qt interval'
    OR 'corrected qt'
    OR 'rate corrected qt'
    OR 'rate-corrected qt'
    OR 'heart rate corrected qt'
  )
):ti,ab,kw

OR

(
  (
    'qt interval'/exp
    OR qt:ti,ab,kw
    OR qtc:ti,ab,kw
    OR qtcf:ti,ab,kw
    OR qtcb:ti,ab,kw
    OR 'qt interval':ti,ab,kw
    OR 'corrected qt':ti,ab,kw
    OR 'rate corrected qt':ti,ab,kw
    OR 'rate-corrected qt':ti,ab,kw
    OR 'heart rate corrected qt':ti,ab,kw
    OR 'qt prolongation':ti,ab,kw
    OR 'cardiac repolarization':ti,ab,kw
    OR 'ventricular repolarization':ti,ab,kw
  )

  AND

  (
    'pharmacokinetics'/exp
    OR 'pharmacodynamics'/exp
    OR 'drug monitoring'/exp
    OR 'area under the curve'/exp
    OR concentration*:ti,ab,kw
    OR exposure:ti,ab,kw
    OR exposures:ti,ab,kw
```

```
  OR 'systemic exposure':ti,ab,kw
  OR cmax:ti,ab,kw
  OR cmin:ti,ab,kw
  OR css:ti,ab,kw
  OR 'maximum concentration':ti,ab,kw
  OR 'peak concentration':ti,ab,kw
  OR 'trough concentration':ti,ab,kw
  OR 'steady state concentration':ti,ab,kw
  OR 'steady-state concentration':ti,ab,kw
  OR 'plasma concentration':ti,ab,kw
  OR 'plasma concentrations':ti,ab,kw
  OR 'serum concentration':ti,ab,kw
  OR 'serum concentrations':ti,ab,kw
  OR 'blood concentration':ti,ab,kw
  OR 'blood concentrations':ti,ab,kw
  OR 'drug concentration':ti,ab,kw
  OR 'drug concentrations':ti,ab,kw
  OR 'plasma level':ti,ab,kw
  OR 'plasma levels':ti,ab,kw
  OR 'serum level':ti,ab,kw
  OR 'serum levels':ti,ab,kw
  OR metabolite*:ti,ab,kw
  OR pharmacokinetic*:ti,ab,kw
  OR pharmacodynamic*:ti,ab,kw
  OR pkpd:ti,ab,kw
  OR 'pk pd':ti,ab,kw
  OR 'pk-pd':ti,ab,kw
  OR 'pharmacokinetic pharmacodynamic':ti,ab,kw
  OR 'pharmacokinetic-pharmacodynamic':ti,ab,kw
  OR auc:ti,ab,kw
  OR 'area under the curve':ti,ab,kw
  OR 'area under the concentration time curve':ti,ab,kw
  OR 'area under the concentration-time curve':ti,ab,kw
)

AND

(
  model*:ti,ab,kw
  OR modelling:ti,ab,kw
  OR modeling:ti,ab,kw
  OR analys*:ti,ab,kw
  OR relationship*:ti,ab,kw
  OR association*:ti,ab,kw
  OR correlate*:ti,ab,kw
  OR correlation*:ti,ab,kw
```

```
      OR regression:ti,ab,kw
      OR slope*:ti,ab,kw
      OR predict*:ti,ab,kw
      OR estimate*:ti,ab,kw
      OR 'linear mixed effect':ti,ab,kw
      OR 'linear mixed effects':ti,ab,kw
      OR 'mixed effect model':ti,ab,kw
      OR 'mixed effects model':ti,ab,kw
      OR 'mixed-effect model':ti,ab,kw
      OR 'mixed-effects model':ti,ab,kw
      OR pharmacometric*:ti,ab,kw
      OR emax:ti,ab,kw
      OR 'e max':ti,ab,kw
      OR nonlinear:ti,ab,kw
      OR 'non linear':ti,ab,kw
      OR 'non-linear':ti,ab,kw
      OR hysteresis:ti,ab,kw
      OR 'effect compartment':ti,ab,kw
      OR 'effect site compartment':ti,ab,kw
      OR 'effect-site compartment':ti,ab,kw
      OR 'exposure response':ti,ab,kw
      OR 'exposure-response':ti,ab,kw
      OR 'concentration response':ti,ab,kw
      OR 'concentration-response':ti,ab,kw
    )
  )

  OR

  (
    'thorough qt':ti,ab,kw
    OR 'thorough qtc':ti,ab,kw
    OR 'thorough qt/qtc':ti,ab,kw
    OR
    (
      tqt:ti,ab,kw
      AND
      (
        qt:ti,ab,kw
        OR qtc:ti,ab,kw
        OR electrocardiogra*:ti,ab,kw
        OR cardiac:ti,ab,kw
      )
    )
  )
)
```

No human, language, publication-type, or publication-year limit will initially be applied in Embase. Animal-only studies will be removed during screening.

3. Scopus

Search interface

Scopus Advanced Search

Search strategy

```
TITLE-ABS-KEY
(
 (
  (
   concentration
   OR exposure
   OR "systemic exposure"
   OR "plasma concentration"
   OR "serum concentration"
   OR "blood concentration"
   OR "drug concentration"
   OR "drug level"
   OR "plasma level"
   OR "serum level"
  )
  W/5
  (
   QT
   OR QTc
   OR QTcF
   OR QTcB
   OR "QT interval"
   OR "corrected QT"
   OR "rate corrected QT"
   OR "rate-corrected QT"
   OR "heart rate corrected QT"
  )
 )

 OR

 (
  (
   QT
   OR QTc
   OR QTcF
   OR QTcB
   OR "QT interval"
```

```
  OR "corrected QT"
  OR "rate corrected QT"
  OR "rate-corrected QT"
  OR "heart rate corrected QT"
  OR "QT prolongation"
  OR "cardiac repolarization"
  OR "ventricular repolarization"
)

AND

(
  concentrat*
  OR exposure
  OR exposures
  OR "systemic exposure"
  OR Cmax
  OR Cmin
  OR Css
  OR "maximum concentration"
  OR "peak concentration"
  OR "trough concentration"
  OR "steady state concentration"
  OR "steady-state concentration"
  OR "plasma concentration"
  OR "serum concentration"
  OR "blood concentration"
  OR "drug concentration"
  OR "plasma level"
  OR "serum level"
  OR metabolite*
  OR pharmacokinetic*
  OR pharmacodynamic*
  OR PKPD
  OR "PK PD"
  OR "PK-PD"
  OR "pharmacokinetic pharmacodynamic"
  OR "pharmacokinetic-pharmacodynamic"
  OR AUC
  OR "area under the curve"
  OR "area under the concentration-time curve"
)

AND

(
```

```
    model*
    OR modelling
    OR modeling
    OR analys*
    OR relationship*
    OR association*
    OR correlate*
    OR correlation*
    OR regression
    OR slope*
    OR predict*
    OR estimate*
    OR "linear mixed effect"
    OR "linear mixed effects"
    OR "mixed effect model"
    OR "mixed effects model"
    OR "mixed-effect model"
    OR "mixed-effects model"
    OR pharmacometric*
    OR Emax
    OR "E-max"
    OR nonlinear
    OR "non-linear"
    OR hysteresis
    OR "effect compartment"
    OR "effect-site compartment"
    OR "exposure response"
    OR "exposure-response"
    OR "concentration response"
    OR "concentration-response"
  )
)

OR

"thorough QT"
OR "thorough QTc"
OR "thorough QT/QTc"
OR
(
  TQT
  AND
  (
    QT
    OR QTc
    OR electrocardiogra*
```

```
      OR cardiac
    )
  )
)
```

No language, publication-year, document-type, or subject-area limits will initially be applied.

4. Cochrane Central Register of Controlled Trials

Search interface

Cochrane Library Search Manager

Search strategy

```
#1 MeSH descriptor: [Pharmacokinetics] explode all trees

#2 MeSH descriptor: [Drug Monitoring] explode all trees

#3 MeSH descriptor: [Dose-Response Relationship, Drug] explode all trees

#4
(
  QT
  OR QTc
  OR QTcF
  OR QTcB
  OR "QT interval"
  OR "corrected QT"
  OR "rate corrected QT"
  OR "rate-corrected QT"
  OR "heart rate corrected QT"
  OR "QT prolongation"
  OR "cardiac repolarization"
  OR "ventricular repolarization"
):ti,ab,kw

#5
(
  concentration*
  OR exposure
  OR exposures
  OR "systemic exposure"
  OR Cmax
  OR Cmin
  OR Css
  OR "maximum concentration"
  OR "peak concentration"
  OR "trough concentration"
```

OR "steady state concentration"
OR "steady-state concentration"
OR "plasma concentration"
OR "serum concentration"
OR "blood concentration"
OR "drug concentration"
OR "plasma level"
OR "serum level"
OR metabolite*
OR pharmacokinetic*
OR pharmacodynamic*
OR PKPD
OR "PK PD"
OR "PK-PD"
OR "pharmacokinetic pharmacodynamic"
OR "pharmacokinetic-pharmacodynamic"
OR AUC
OR "area under the curve"
OR "area under the concentration-time curve"
):ti,ab,kw

#6 #1 OR #2 OR #5

#7
(
model*
OR modelling
OR modeling
OR analys*
OR relationship*
OR association*
OR correlate*
OR correlation*
OR regression
OR slope*
OR predict*
OR estimate*
OR "linear mixed effect"
OR "linear mixed effects"
OR "mixed effect model"
OR "mixed effects model"
OR pharmacometric*
OR Emax
OR nonlinear
OR "non-linear"
OR hysteresis

```
  OR "effect compartment"
  OR "effect-site compartment"
  OR "exposure response"
  OR "exposure-response"
  OR "concentration response"
  OR "concentration-response"
):ti,ab,kw

#8 #3 OR #7

#9
(
  (
    concentration
    OR exposure
    OR "systemic exposure"
    OR "plasma concentration"
    OR "serum concentration"
    OR "drug concentration"
  )
  NEAR/5
  (
    QT
    OR QTc
    OR QTcF
    OR QTcB
    OR "QT interval"
    OR "corrected QT"
  )
):ti,ab,kw

#10
(
  "thorough QT"
  OR "thorough QTc"
  OR "thorough QT/QTc"
  OR
  (
    TQT
    AND
    (
      QT
      OR QTc
      OR electrocardiogra*
      OR cardiac
    )
```

)
):ti,ab,kw

#11 #9 OR (#4 AND #6 AND #8) OR #10

The results will be restricted to the CENTRAL database when the final search is executed.

5. ClinicalTrials.gov

Search interface

ClinicalTrials.gov—Other Terms or Expert Search

Because trial registry records frequently provide limited methodological detail, several shorter searches will be run separately and the results will be combined and deduplicated.

Search 1: Direct concentration–QT terminology

```
"concentration-QT" OR "concentration-QTc" OR
"exposure-QT" OR "exposure-QTc" OR
"concentration QT" OR "concentration QTc" OR
"exposure QT" OR "exposure QTc"
```

Search 2: QT combined with pharmacokinetic exposure

```
(
 "QT interval"
 OR QTc
 OR QTcF
 OR QTcB
 OR "corrected QT"
 OR "QT prolongation"
)
AND
(
 concentration
 OR exposure
 OR pharmacokinetic
 OR pharmacokinetics
 OR PK
 OR PKPD
 OR Cmax
 OR metabolite
 OR "plasma concentration"
 OR "serum concentration"
)
```

Search 3: Thorough QT studies

```
"thorough QT" OR "thorough QTc" OR "thorough QT/QTc"
```

Search 4: TQT abbreviation

```
TQT AND
(
  QT
  OR QTc
  OR electrocardiogram
  OR electrocardiography
  OR cardiac
)
```

Search 5: Repolarisation terminology

```
(
  "cardiac repolarization"
  OR "cardiac repolarisation"
  OR "ventricular repolarization"
  OR "ventricular repolarisation"
)
AND
(
  concentration
  OR exposure
  OR pharmacokinetic
  OR pharmacokinetics
  OR Cmax
  OR metabolite
)
```

No restriction will be applied according to recruitment status, study completion status, study phase, country, age, or date. Both interventional and observational studies will be considered.

Registry records will be used to identify completed, ongoing, unpublished, and potentially relevant studies. Published reports linked to eligible registry records will be sought separately.

6. Google Scholar

Google Scholar does not support the same reproducible field-based and proximity syntax as bibliographic databases. Therefore, several short exact-phrase searches will be run separately.

Search 1

"concentration-QT"

Search 2

"concentration-QTc"

Search 3

"exposure-QT"

Search 4

"exposure-QTc"

Search 5

"concentration response" QTc drug

Search 6

"exposure response" QTc drug

Search 7

"pharmacokinetic pharmacodynamic" QTc

Search 8

"PK-PD" QTc concentration

Search 9

"thorough QT" concentration

Search 10

"effect compartment" QTc concentration

Search 11

hysteresis QTc concentration

Search 12

"active metabolite" QTc concentration

For each Google Scholar query, the first 200 results ranked by relevance will be screened. The search date, exact query, number of results displayed, and number of results screened will be recorded. The searches will be performed in a logged-out browser or a private browsing window where feasible to reduce the influence of personalised search history.

Google Scholar will be treated as a supplementary source for identifying grey literature, theses, conference materials, citation-linked publications, and records not retrieved from the principal bibliographic databases.

Additional search methods

The reference lists of all included studies and relevant reviews will be manually examined. Forward citation searching will be undertaken using Scopus and Google Scholar. Related publications, supplementary materials, regulatory reports, and linked trial registry records will be examined when necessary to obtain additional concentration–QT/QTc data.

Search results from all sources will be exported to reference-management software and deduplicated before screening. Potential duplicate reports derived from the same clinical study will be linked and treated as a single underlying study, while complementary data may be extracted from multiple reports.
