# Supplementary material for: Clinical pharmacokinetic-pharmacodynamic relationships between drug exposure and the QT/QTc interval: Protocol for a systematic review and quantitative synthesis
Source: PLoS One. 2026 Oct 28;21(10):e0357131. doi: 10.1371/journal.pone.0357131 (PMC13716211; doi:10.1371/journal.pone.0357131)
Supplement: Supplementary file 2 [file pone.0357131.s005.pdf]

PONE-D-26-40572

Clinical Pharmacokinetic-Pharmacodynamic Relationships between Drug Exposure and the QT/QTc Interval: Protocol for a Systematic Review and Quantitative Synthesis

PLOS One

Journal Requirements:

When submitting your revision, we need you to address these additional requirements.

1. Please ensure that your manuscript meets PLOS ONE's style requirements, including those for file naming. The PLOS ONE style templates can be found at https://journals.plos.org/plosone/s/file?id=wjVg/PLOSOne_formatting_sample_main_body.pdf and https://journals.plos.org/plosone/s/file?id=ba62/PLOSOne_formatting_sample_title_authors_affiliations.pdf

Response: *Thank you. We have reviewed the revised manuscript and all accompanying files for compliance with the PLOS ONE formatting and file-naming requirements. The clean manuscript, marked-up manuscript, figure file, and Supporting Information files have been prepared accordingly.*

2. When completing the data availability statement of the submission form, you indicated that you will make your data available on acceptance. We strongly recommend all authors decide on a data sharing plan before acceptance, as the process can be lengthy and hold up publication timelines. Please note that, though access restrictions are acceptable now, your entire data will need to be made freely accessible if your manuscript is accepted for publication. This policy applies to all data except where public deposition would breach compliance with the protocol approved by your research ethics board. If you are unable to adhere to our open data policy, please kindly revise your statement to explain your reasoning and we will seek the editor's input on an exemption. Please be assured that, once you have provided your new statement, the assessment of your exemption will not hold up the peer review process.

Response: *Thank you for this clarification. We have revised the Data Availability Statement to accurately reflect the nature of the present article as a study protocol. No datasets have yet been generated or analysed for this protocol manuscript.*

3. Please upload a new copy of Figure 1 as the detail is not clear. Please follow the link for more information: https://journals.plos.org/plosone/s/figures.

Response: *Thank you. Figure 1 has been revised to improve clarity and readability. The text size and visual elements have been optimized, and a new high-resolution version of the figure has been uploaded in accordance with the PLOS ONE figure specifications. The scientific content of the figure has not changed.*

4. If the reviewer comments include a recommendation to cite specific previously published works, please review and evaluate these publications to determine whether they are relevant and should be cited. There is no requirement to cite these works unless the editor has indicated otherwise.

Response: *We have evaluated the literature relevant to the reviewer's comments and have added relevant references where they directly support the revised manuscript. No reference was added solely because of a citation recommendation.*

5. Please review your reference list to ensure that it is complete and correct. If you have cited papers that have been retracted, please include the rationale for doing so in the manuscript text, or remove these references and replace them with relevant current references. Any changes to the reference list should be mentioned in the rebuttal letter that accompanies your revised manuscript. If you need to cite a retracted article, indicate the article's retracted status in the References list and also include a citation and full reference for the retraction notice.

Response: *Thank you. We have carefully reviewed the entire reference list for completeness and bibliographic accuracy, including author names, article titles, journal information, publication year, pagination/article numbers, and DOI information. We also checked the cited literature for retraction status. Relevant corrections and newly added references are reflected in the revised manuscript.*

Additional Editor Comments:

The proposal is well written with clear objectives and methods. However, there are a few comments from our reviewer which you need to address, and highlight the changes in your amended protocol.

Reviewer's Responses to Questions

Comments to the Author

1. Does the manuscript provide a valid rationale for the proposed study, with clearly identified and justified research questions?

The research question outlined is expected to address a valid academic problem or topic and contribute to the base of knowledge in the field.

Reviewer #1: Yes

---

2. Is the protocol technically sound and planned in a manner that will lead to a meaningful outcome and allow testing the stated hypotheses?

The manuscript should describe the methods in sufficient detail to prevent undisclosed flexibility in the experimental procedure or analysis pipeline, including sufficient outcome-neutral conditions (e.g. necessary controls, absence of floor or ceiling effects) to test the proposed hypotheses and a statistical power analysis where applicable. As there may be aspects of the methodology and analysis which can only be refined once the work is undertaken, authors should outline potential assumptions and explicitly describe what aspects of the proposed analyses, if any, are exploratory.

Reviewer #1: Yes

---

3. Is the methodology feasible and described in sufficient detail to allow the work to be replicable?

Descriptions of methods and materials in the protocol should be reported in sufficient detail for another researcher to reproduce all experiments and analyses. The protocol should describe the appropriate controls, sample size calculations, and replication needed to ensure that the data are robust and reproducible.

Reviewer #1: Yes

---

4. Have the authors described where all data underlying the findings will be made available when the study is complete?

The PLOS Data policy requires authors to make all data underlying the findings described in their manuscript fully available without restriction, with rare exception, at the time of publication. The data should be provided as part of the manuscript or its supporting information, or deposited to a public repository. For example, in addition to summary statistics, the data points behind means, medians and variance measures should be available. If there are restrictions on publicly sharing data—e.g. participant privacy or use of data from a third party—those must be specified.

Reviewer #1: Yes

---

5. Is the manuscript presented in an intelligible fashion and written in standard English?

PLOS ONE does not copyedit accepted manuscripts, so the language in submitted articles must be clear, correct, and unambiguous. Any typographical or grammatical errors should be corrected at revision, so please note any specific errors here.

Reviewer #1: Yes

6. Review Comments to the Author

Reviewer #1: 1. The authors are encouraged to provide a brief clarification regarding the novelty of the present systematic review compared with previously published methodological reviews on concentration-QT/QTc modelling. This would help readers better understand the specific knowledge gap that this review intends to address.

Response: *Thank you for this helpful suggestion. We have expanded the Introduction to more explicitly distinguish the present systematic review from previous methodological reviews, regulatory perspectives, and practical tutorials on concentration–QT/QTc modelling.*

2. The eligibility criteria are generally well described; however, the authors may consider adding a few practical examples of eligible and non-eligible studies, particularly regarding studies using Cmax/AUC-based exposure metrics versus direct concentration-QT/QTc models, to improve transparency during study selection.

Response: *We agree and have added practical operational examples immediately following the eligibility criteria. We now explicitly distinguish direct time-matched concentration–QT/QTc analyses from studies using continuous summary exposure measures such as Cmax, Cmin, or AUC. We also clarify that analyses based solely on dose groups, categorical exposure strata, or simulation-only exposure profiles without original human concentration–QT/QTc data are not eligible. This addition is intended to improve reproducibility of study selection.*

3. The authors may consider clarifying how disagreements between reviewers will be managed during data extraction when multiple eligible models or multiple QT correction methods are reported within the same study.

Response: *We have clarified the adjudication procedure for studies reporting multiple eligible models or QT correction methods. Both reviewers will independently extract all eligible analyses and apply the prespecified hierarchy. Alternative models and correction methods will be retained rather than discarded, while the prespecified primary estimate will be selected for the principal synthesis. Any disagreement regarding model classification or selection will be resolved by consensus or third-reviewer adjudication and documented in the review decision log.*

4. The manuscript describes several possible model structures (linear, nonlinear, effect-compartment, and joint parent-metabolite models). The authors may consider providing additional details on how model-specific parameters will be compared or summarized when direct quantitative pooling is not feasible.

Response: *We agree that the handling of heterogeneous model structures required further specification. We have expanded the Data synthesis section to describe model-specific synthesis*

explicitly. Linear slopes, E_{max}/EC_{50} parameters, sigmoid E_{max} parameters, delayed-effect parameters, and parent–metabolite model estimates will be retained in their native parameterisation. Structurally different models will not be forced into a common linear effect measure. When quantitative pooling is not justified, estimates will be grouped by active moiety, estimand, and model class and summarised using structured tables and SWiM principles. Comparable model-predicted QT/QTc effects at clinically relevant exposures will also be considered as clinically interpretable summaries where appropriate.

5. The concentration-QT/QTc-specific methodological appraisal framework is a useful component of this protocol. The authors may clarify whether this framework has been previously applied or whether it was specifically developed for this review, and how its reliability between reviewers will be assessed.

Response: *We have clarified that the concentration–QT/QTc-specific framework was developed specifically for this review and is not presented as a previously validated standalone risk-of-bias instrument. It supplements established design-based tools and was informed by ICH E14/S7B and concentration-QTc pharmacometric guidance. We have also added a prespecified calibration and reliability procedure. Two reviewers will independently pilot the framework on methodologically diverse studies before formal application, and initial domain-level agreement will be documented using percentage agreement and, where estimable, Cohen's κ with 95%CI. Disagreements will subsequently be resolved by consensus or third-reviewer adjudication.*

6. The authors have appropriately planned sensitivity analyses; however, they may consider explicitly defining which analyses will be considered exploratory in the final review to facilitate appropriate interpretation of the findings.

Response: *We have added an explicit distinction between primary, supportive sensitivity, and exploratory analyses. Meta-regression, heterogeneity-driven subgroup analyses, molar-scale presentations, analyses of non-primary models, estimates requiring substantial reconstruction, and any unplanned analyses prompted by the observed evidence will be identified as exploratory. Any post hoc analysis will be labelled explicitly in the final review and will not be interpreted as confirmatory.*
