# Supplementary material for: Clinical pharmacokinetic-pharmacodynamic relationships between drug exposure and the QT/QTc interval: Protocol for a systematic review and quantitative synthesis
Source: PLoS One. 2026 Oct 28;21(10):e0357131. doi: 10.1371/journal.pone.0357131 (PMC13716211; doi:10.1371/journal.pone.0357131)
Supplement: Supplementary file 3 [file pone.0357131.s001.docx]

PRISMA-P 2015 checklist

Manuscript: Clinical pharmacokinetic–pharmacodynamic relationships between drug exposure and the QT/QTc interval: protocol for a systematic review and quantitative synthesis

| Item | PRISMA-P requirement | Location in manuscript |
| --- | --- | --- |
| 1a | Identify the report as a protocol of a systematic review | Title page |
| 1b | Identify as an update, if applicable | Not applicable; first review |
| 2 | Registration information | Title page; Registration and protocol amendments |
| 3a | Names, affiliations, email and mailing address | Title page; full mailing address to be added before submission |
| 3b | Author contributions and guarantor | Author contributions |
| 4 | Protocol amendments | Registration and protocol amendments |
| 5a | Sources of financial or other support | Funding |
| 5b | Sponsor/funder | Funding |
| 5c | Role of sponsor/funder | Funding; not applicable |
| 6 | Rationale | Introduction |
| 7 | Objectives and review questions | Review objectives |
| 8 | Eligibility criteria | Eligibility criteria; Table 1 |
| 9 | Information sources | Information sources |
| 10 | Draft search strategy | Search strategy; S2 File |
| 11a | Data management | Data management |
| 11b | Selection process | Study selection |
| 11c | Data collection process | Data collection process |
| 12 | Data items and assumptions | Data items; Table 2 |
| 13 | Outcomes and prioritisation | Outcomes and prioritisation; Table 3 |
| 14 | Risk of bias in individual studies | Risk of bias and methodological appraisal; Table 4 |
| 15a | Criteria for quantitative synthesis | Criteria for quantitative synthesis |
| 15b | Summary measures and methods of combining data | Effect measures and harmonisation; Meta-analysis model |
| 15c | Additional analyses | Exploration of heterogeneity; Sensitivity analyses |
| 15d | Type of summary if meta-analysis is inappropriate | Descriptive and structured synthesis |
| 16 | Assessment of meta-biases | Assessment of reporting bias and missing evidence |
| 17 | Assessment of confidence in cumulative evidence | Certainty of evidence |
