# Supplementary material for: Clinical pharmacokinetic-pharmacodynamic relationships between drug exposure and the QT/QTc interval: Protocol for a systematic review and quantitative synthesis
Source: PLoS One. 2026 Oct 28;21(10):e0357131. doi: 10.1371/journal.pone.0357131 (PMC13716211; doi:10.1371/journal.pone.0357131)
Supplement: Supplementary file 4 [file pone.0357131.s003.docx]

**S3 File**

**Draft data-extraction form and concentration–QT/QTc methodological appraisal form**

*Clinical Pharmacokinetic–Pharmacodynamic Relationships Between Drug Exposure and QT/QTc Interval: A Systematic Review and Quantitative Synthesis*

**Purpose and status.** This supplement provides the prespecified draft forms to be piloted before formal extraction. The concentration–QT/QTc appraisal component is a review-specific methodological framework informed by regulatory and pharmacometric principles; it is not presented as a previously validated standalone risk-of-bias instrument. Design-based risk-of-bias tools (RoB 2, ROBINS-I, and ROBINS-E as applicable) will be applied separately.

# Part A. Data-extraction form

## A1. Study identification and report linkage

| **Variable** | **Extracted value** | **Guidance / permitted coding** |
| --- | --- | --- |
| Unique study-family ID |  |  |
| Report ID / citation |  |  |
| First author / year |  |  |
| Country / centres |  |  |
| Sponsor / funding |  |  |
| Registry identifier |  |  |
| Related reports / supplements / regulatory documents |  |  |
| Publication status |  | Published / unpublished / abstract / regulatory report / other |
| Reviewer / extraction date |  |  |

## A2. Study design and participants

| **Variable** | **Extracted value** | **Guidance / permitted coding** |
| --- | --- | --- |
| Study design |  | Randomised / non-randomised / crossover / parallel / TQT / SAD / MAD / DDI / organ impairment / observational PK-PD / pooled analysis / other |
| Study phase |  | Phase I / II / III / IV / not stated |
| Setting |  | Healthy-volunteer unit / hospital / outpatient / other |
| Population |  | Healthy volunteers / patients; condition if applicable |
| Sample size analysed for C-QT/QTc |  |  |
| Age |  | Mean/median, dispersion, range |
| Sex distribution |  |  |
| Ethnicity / race if reported |  |  |
| Key inclusion/exclusion criteria |  |  |
| Baseline QT/QTc and heart rate |  |  |
| Baseline cardiac risk / concomitant QT-risk factors |  |  |

## A3. Drug, dosing, and exposure context

| **Variable** | **Extracted value** | **Guidance / permitted coding** |
| --- | --- | --- |
| Parent drug |  |  |
| Active / relevant metabolite(s) |  |  |
| Therapeutic class |  |  |
| Route of administration |  |  |
| Dose(s) / regimen(s) |  |  |
| Single vs multiple dose |  |  |
| Therapeutic / supratherapeutic exposure |  |  |
| Steady state achieved? |  | Yes / No / unclear |
| Exposure-modifying condition |  | DDI / renal impairment / hepatic impairment / accumulation / food / genotype / other |
| Comparator / placebo / positive control |  |  |
| Moxifloxacin or other assay-sensitivity control |  | If applicable |

## A4. Pharmacokinetic measurements

| **Variable** | **Extracted value** | **Guidance / permitted coding** |
| --- | --- | --- |
| Biological matrix |  | Plasma / serum / whole blood / other |
| Analyte(s) measured |  | Parent / metabolite / both |
| Bioanalytical method |  | LC-MS/MS / other; validation details |
| Lower limit of quantification |  |  |
| PK sampling schedule |  |  |
| ECG–PK temporal matching |  | Exact / nominal / model-linked / unclear |
| Observed vs model-predicted concentration |  |  |
| Exposure metric used in analysis |  | Time-matched concentration / Cmax / Cmin / Css / AUC / other |
| Concentration unit |  |  |
| Exposure range studied |  | Min/max or relevant percentiles |
| Clinically relevant reference exposure |  | Therapeutic Cmax, steady-state Cmax, maximum clinical exposure, or specified alternative |

## A5. ECG and QT/QTc assessment

| **Variable** | **Extracted value** | **Guidance / permitted coding** |
| --- | --- | --- |
| ECG acquisition method |  | 12-lead / continuous / Holter / other |
| Device / digital system |  |  |
| ECG replicate strategy |  | Single / triplicate / other |
| ECG timing relative to dosing and PK sampling |  |  |
| Central vs local reading |  |  |
| Reader blinding |  | Yes / No / unclear |
| QT measurement approach |  | Manual / automated / semi-automated |
| QT correction method |  | QTcF / QTcB / QTcI / study-specific / other |
| Drug effect on heart rate assessed? |  | Yes / No; method/result |
| Baseline correction |  | Yes / No; method |
| Placebo adjustment |  | Yes / No; method |
| Nominal-time / circadian effect handled? |  | Yes / No; method |

## A6. Concentration–QT/QTc model

| **Variable** | **Extracted value** | **Guidance / permitted coding** |
| --- | --- | --- |
| Primary QT/QTc endpoint |  | QT / QTcF / QTcB / QTcI / ΔQTc / ΔΔQTc / other |
| Model type |  | Linear mixed effects / Emax / sigmoid Emax / nonlinear / effect compartment / indirect response / other |
| Dependent variable |  |  |
| Exposure variable |  |  |
| Random effects / covariance structure |  |  |
| Prespecified primary model? |  | Yes / No / unclear |
| Treatment/time/baseline covariates |  |  |
| Parent and metabolite modeled jointly? |  | Yes / No / not applicable |
| Linearity assessed? |  | Yes / No; method |
| Hysteresis / delayed effect assessed? |  | Yes / No; method |
| Alternative models explored? |  |  |
| Software / version |  |  |
| Model diagnostics reported |  | Residuals / GOF / VPC / bootstrap / sensitivity / other |

## A7. Quantitative results and uncertainty

| **Variable** | **Extracted value** | **Guidance / permitted coding** |
| --- | --- | --- |
| Concentration–QT/QTc slope |  | Estimate, unit |
| Slope SE / CI / credible interval |  |  |
| Intercept / treatment effect at zero concentration |  |  |
| Predicted ΔQTc or ΔΔQTc at therapeutic exposure |  | Estimate and uncertainty |
| Predicted effect at high / worst-case clinical exposure |  | Estimate and uncertainty |
| Upper bound of 90% CI at clinically relevant exposure |  |  |
| 10-ms regulatory threshold excluded? |  | Yes / No / not assessable |
| C5 (concentration for +5 ms QTc) |  | Reported / derivable / not available |
| C10 (concentration for +10 ms QTc) |  | Reported / derivable / not available |
| Nonlinear model parameters |  | Emax, EC50, Hill coefficient, etc., if applicable |
| Metabolite-specific effect estimate |  |  |
| Any negative/flat concentration–QT relationship |  |  |
| Data derivation performed by reviewers |  | Formula / conversion / digitisation / none |
| Notes on extrapolation |  | No unsupported extrapolation beyond observed/model-supported range |

## A8. Additional methodological and reporting data

| **Variable** | **Extracted value** | **Guidance / permitted coding** |
| --- | --- | --- |
| Missing concentration/ECG observations |  | Extent and handling |
| Outliers |  | Definition and handling |
| BLQ concentrations |  | Handling |
| Protocol deviations |  | Handling |
| Sensitivity analyses |  |  |
| Subgroups / covariate analyses |  |  |
| Selective model/result reporting suspected? |  | Yes / No / unclear; rationale |
| Author contacted for clarification? |  | Date / response / data obtained |
| Reviewer comments |  |  |

# Part B. Concentration–QT/QTc methodological appraisal form

**Rating categories:** Low risk / Some concerns / High risk / Insufficient information. The judgment should reflect the likely impact on the specific concentration–QT/QTc result or estimand being extracted, not merely the overall study. No numerical total score will be calculated.

| **Domain** | **Signalling question** | **Key considerations** | **Judgment** | **Direction of bias (if inferable)** | **Rationale / supporting text** |
| --- | --- | --- | --- | --- | --- |
| 1. Study design and participant selection | Were the design, population, exclusions, and analysis set appropriate for estimating the intended exposure–QT/QTc relationship without important selection bias? | Eligibility/analysis set; exclusions after dosing; crossover/parallel appropriateness; enrichment; baseline cardiac characteristics. | [ ] Low [ ] Some concerns [ ] High [ ] Insufficient information | [ ] Toward larger QT effect [ ] Toward smaller QT effect [ ] Unpredictable [ ] Not inferable |  |
| 2. Adequacy of dose and exposure range | Did the study generate a sufficiently broad and clinically relevant systemic exposure range, including clinically relevant high exposure when required for the study objective? | Therapeutic vs supratherapeutic range; exposure overlap; maximum clinical exposure; organ impairment/DDI exposure. | [ ] Low [ ] Some concerns [ ] High [ ] Insufficient information | [ ] Toward larger QT effect [ ] Toward smaller QT effect [ ] Unpredictable [ ] Not inferable |  |
| 3. PK sampling and bioanalytical validity | Were concentration measurements sufficiently accurate, precise, and timed to support the exposure–QT/QTc analysis? | Validated assay; LLOQ; sample handling; sampling density; missing PK; observed vs model-predicted concentrations. | [ ] Low [ ] Some concerns [ ] High [ ] Insufficient information | [ ] Toward larger QT effect [ ] Toward smaller QT effect [ ] Unpredictable [ ] Not inferable |  |
| 4. ECG acquisition and QT measurement quality | Were ECG acquisition, replicate strategy, QT measurement, reading process, and blinding adequate to minimise measurement bias? | 12-lead/digital methods; replicates; central reading; manual/automated method; reader blinding; ECG quality control. | [ ] Low [ ] Some concerns [ ] High [ ] Insufficient information | [ ] Toward larger QT effect [ ] Toward smaller QT effect [ ] Unpredictable [ ] Not inferable |  |
| 5. Temporal matching of ECG and PK | Were ECG measurements and concentrations appropriately time-matched or linked by a justified PK/PD model? | Exact paired times; nominal-time mismatch; interpolation/model linkage; exposure changes between sampling and ECG. | [ ] Low [ ] Some concerns [ ] High [ ] Insufficient information | [ ] Toward larger QT effect [ ] Toward smaller QT effect [ ] Unpredictable [ ] Not inferable |  |
| 6. Baseline, placebo, and time-effect control | Were baseline QT/QTc, placebo effects, nominal-time/circadian effects, and other systematic temporal effects appropriately handled? | ΔQTc/ΔΔQTc; baseline adjustment; placebo adjustment; time factor; period/sequence effects for crossover designs. | [ ] Low [ ] Some concerns [ ] High [ ] Insufficient information | [ ] Toward larger QT effect [ ] Toward smaller QT effect [ ] Unpredictable [ ] Not inferable |  |
| 7. Heart-rate correction and drug-related HR effects | Was the QT correction method appropriate, and was any drug-related heart-rate effect evaluated sufficiently to support the chosen correction? | QTcF/QTcI vs QTcB; HR change; correction independence; study-specific QTc derivation. | [ ] Low [ ] Some concerns [ ] High [ ] Insufficient information | [ ] Toward larger QT effect [ ] Toward smaller QT effect [ ] Unpredictable [ ] Not inferable |  |
| 8. Parent drug and metabolite handling | Were relevant active/QT-active metabolites measured and modeled when they could materially contribute to the QT/QTc effect? | Metabolite exposure; correlated parent/metabolite concentrations; joint model; omission of plausible active metabolite. | [ ] Low [ ] Some concerns [ ] High [ ] Insufficient information | [ ] Toward larger QT effect [ ] Toward smaller QT effect [ ] Unpredictable [ ] Not inferable |  |
| 9. Model prespecification and structural appropriateness | Was the concentration–QT/QTc model prespecified or otherwise adequately justified, and was its structural form suitable for the data and estimand? | Linear mixed model assumptions; covariates; random effects; intercept; treatment effect; model selection process. | [ ] Low [ ] Some concerns [ ] High [ ] Insufficient information | [ ] Toward larger QT effect [ ] Toward smaller QT effect [ ] Unpredictable [ ] Not inferable |  |
| 10. Nonlinearity, hysteresis, and delayed effects | Were departures from a direct linear relationship evaluated when suggested by pharmacology, plots, timing, or diagnostics? | Nonlinear alternatives; effect compartment; hysteresis loops; delayed metabolite effect; time delay. | [ ] Low [ ] Some concerns [ ] High [ ] Insufficient information | [ ] Toward larger QT effect [ ] Toward smaller QT effect [ ] Unpredictable [ ] Not inferable |  |
| 11. Missing data, outliers, BLQ values, and protocol deviations | Were missing observations, influential outliers, BLQ concentrations, and major deviations handled transparently and appropriately? | Exclusion rules; imputation; robust analyses; sensitivity analyses; reasons for missing paired data. | [ ] Low [ ] Some concerns [ ] High [ ] Insufficient information | [ ] Toward larger QT effect [ ] Toward smaller QT effect [ ] Unpredictable [ ] Not inferable |  |
| 12. Model diagnostics and statistical uncertainty | Were model fit, diagnostics, uncertainty, and sensitivity analyses sufficient to support the reported effect estimate? | Residual plots; GOF; bootstrap/VPC; SE/CI; shrinkage if relevant; alternative structures; influence analyses. | [ ] Low [ ] Some concerns [ ] High [ ] Insufficient information | [ ] Toward larger QT effect [ ] Toward smaller QT effect [ ] Unpredictable [ ] Not inferable |  |
| 13. Selective reporting of analyses or results | Is there evidence that model structures, exposure ranges, metabolites, time points, or uncertainty measures were selectively reported based on results? | Protocol/registry comparison; multiple models with selective presentation; omitted negative analyses; incomplete CI/SE reporting. | [ ] Low [ ] Some concerns [ ] High [ ] Insufficient information | [ ] Toward larger QT effect [ ] Toward smaller QT effect [ ] Unpredictable [ ] Not inferable |  |

## Overall concentration–QT/QTc methodological judgment

| **Overall judgment** | [ ] Low risk [ ] Some concerns [ ] High risk [ ] Insufficient information |
| --- | --- |
| **Domains driving overall judgment** |  |
| **Likely direction and magnitude of impact on the principal estimand** |  |
| **Reviewer rationale** |  |

# Part C. Reviewer reconciliation and study-family rules

- Extraction and appraisal will be performed independently by two reviewers after piloting the forms on a small sample of eligible studies.
- Multiple publications from the same underlying clinical study will be linked as one study family. Complementary information may be extracted across reports, but participants will not be double-counted.
- When multiple eligible C–QT/QTc models are reported, the prespecified primary model will be prioritised. If no primary model is identified, a predefined hierarchy based on the review estimand, QT correction, clinical relevance of exposure, and model adequacy will be applied and documented.
- Placebo-adjusted, baseline-corrected QTcF (ΔΔQTcF) will be prioritised when multiple comparable QT correction outcomes are available, while alternative correction methods will be retained for sensitivity/secondary analyses.
- Concentration units may be converted within the same drug/analyte when the conversion is exact. Raw slopes will not be pooled indiscriminately across different drugs.
- C5/C10 values will be extracted if reported or derived only when derivation is mathematically justified within the observed or model-supported exposure range; unsupported extrapolation will not be performed.
- Disagreements will be resolved by consensus or a third reviewer, and all post-pilot changes to the form will be versioned and documented.

# References informing the appraisal framework

International Council for Harmonisation. E14/S7B Questions and Answers: Clinical and Nonclinical Evaluation of QT/QTc Interval Prolongation and Proarrhythmic Potential. Step 4. 2022.

Garnett C, Bonate PL, Dang Q, Ferber G, Huang D, Liu J, et al. Scientific white paper on concentration-QTc modeling. J Pharmacokinet Pharmacodyn. 2018;45(3):383-397. doi:10.1007/s10928-017-9558-5.

Sterne JAC, Savović J, Page MJ, Elbers RG, Blencowe NS, Boutron I, et al. RoB 2: a revised tool for assessing risk of bias in randomised trials. Cochrane Methods; current version.

Sterne JA, Hernán MA, Reeves BC, Savović J, Berkman ND, Viswanathan M, et al. ROBINS-I: a tool for assessing risk of bias in non-randomised studies of interventions. BMJ. 2016;355:i4919. doi:10.1136/bmj.i4919.
