# Supplementary material for: Clinical pharmacokinetic-pharmacodynamic relationships between drug exposure and the QT/QTc interval: Protocol for a systematic review and quantitative synthesis
Source: PLoS One. 2026 Oct 28;21(10):e0357131. doi: 10.1371/journal.pone.0357131 (PMC13716211; doi:10.1371/journal.pone.0357131)
Supplement: Peer review history

# Peer review: Decision Letter 0

**DOI:** 10.1371/journal.pone.0357131.r001

**Authors:** Sarah Nanzigu

24 Sep 2026

Dear Dr.  Mohammadnezhad,

Thank you for submitting your manuscript to PLOS One. After careful consideration, we feel that it has merit but does not fully meet PLOS One’s publication criteria as it currently stands. Therefore, we invite you to submit a revised version of the manuscript that addresses the points raised during the review process.

Please submit your revised manuscript by Oct 09 2026 11:59PM. If you will need more time than this to complete your revisions, please reply to this message or contact the journal office at plosone@plos.org. When you're ready to submit your revision, log on to https://www.editorialmanager.com/pone/ and select the 'Submissions Needing Revision' folder to locate your manuscript file.

- A letter that responds to each point raised by the academic editor and reviewer(s). You should upload this letter as a separate file labeled 'Response to Reviewers'.
- A marked-up copy of your manuscript that highlights changes made to the original version. You should upload this as a separate file labeled 'Revised Manuscript with Track Changes'.
- An unmarked version of your revised paper without tracked changes. You should upload this as a separate file labeled 'Manuscript'.

If you would like to make changes to your financial disclosure, please include your updated statement in your cover letter. Guidelines for resubmitting your figure files are available below the reviewer comments at the end of this letter.

If applicable, we recommend that you deposit your laboratory protocols in protocols.io to enhance the reproducibility of your results. Protocols.io assigns your protocol its own identifier (DOI) so that it can be cited independently in the future. For instructions see: https://journals.plos.org/plosone/s/submission-guidelines#loc-laboratory-protocols. Additionally, PLOS One offers an option for publishing peer-reviewed Lab Protocol articles, which describe protocols hosted on protocols.io. Read more information on sharing protocols at https://plos.org/protocols?utm_medium=editorial-email&utm_source=authorletters&utm_campaign=protocols.

As the corresponding author, your ORCID iD is verified in the submission system and will appear in the published article. PLOS supports the use of ORCID, and we encourage all coauthors to register for an ORCID iD and use it as well. Please encourage your coauthors to verify their ORCID iD within the submission system before final acceptance, as unverified ORCID iDs will not appear in the published article. *Only*  the individual author can complete the verification step; PLOS staff *cannot*  verify ORCID iDs on behalf of authors.

We look forward to receiving your revised manuscript.

Kind regards,

Sarah Nanzigu, Ph.D.,MSc.,MBchB

Academic Editor

PLOS One

Journal Requirements:

When submitting your revision, we need you to address these additional requirements.

1.Please ensure that your manuscript meets PLOS ONE's style requirements, including those for file naming. The PLOS ONE style templates can be found at

https://journals.plos.org/plosone/s/file?id=wjVg/PLOSOne_formatting_sample_main_body.pdf and

https://journals.plos.org/plosone/s/file?id=ba62/PLOSOne_formatting_sample_title_authors_affiliations.pdf

2. When completing the data availability statement of the submission form, you indicated that you will make your data available on acceptance. We strongly recommend all authors decide on a data sharing plan before acceptance, as the process can be lengthy and hold up publication timelines. Please note that, though access restrictions are acceptable now, your entire data will need to be made freely accessible if your manuscript is accepted for publication. This policy applies to all data except where public deposition would breach compliance with the protocol approved by your research ethics board. If you are unable to adhere to our open data policy, please kindly revise your statement to explain your reasoning and we will seek the editor's input on an exemption. Please be assured that, once you have provided your new statement, the assessment of your exemption will not hold up the peer review process.

3. Please upload a new copy of Figure 1 as the detail is not clear. Please follow the link for more information:  https://journals.plos.org/plosone/s/figures.

4. If the reviewer comments include a recommendation to cite specific previously published works, please review and evaluate these publications to determine whether they are relevant and should be cited. There is no requirement to cite these works unless the editor has indicated otherwise.

5. Please review your reference list to ensure that it is complete and correct. If you have cited papers that have been retracted, please include the rationale for doing so in the manuscript text, or remove these references and replace them with relevant current references. Any changes to the reference list should be mentioned in the rebuttal letter that accompanies your revised manuscript. If you need to cite a retracted article, indicate the article’s retracted status in the References list and also include a citation and full reference for the retraction notice.

Additional Editor Comments:

The proposal is well written with clear objectives and methods. However, there are a few comments from our reviewer which you need to address, and highlight the changes in your amended protocol.

[Note: HTML markup is below. Please do not edit.]

Reviewer's Responses to Questions

**Comments to the Author**

1. Does the manuscript provide a valid rationale for the proposed study, with clearly identified and justified research questions?

Reviewer #1: Yes

**********

2. Is the protocol technically sound and planned in a manner that will lead to a meaningful outcome and allow testing the stated hypotheses?

Reviewer #1: Yes

**********

3. Is the methodology feasible and described in sufficient detail to allow the work to be replicable?

Reviewer #1: Yes

**********

4. Have the authors described where all data underlying the findings will be made available when the study is complete?

The PLOS Data policy

Reviewer #1: Yes

**********

5. Is the manuscript presented in an intelligible fashion and written in standard English?

Reviewer #1: Yes

**********

Please use the space provided to explain your answers to the questions above and, if applicable, provide comments about issues authors must address before this protocol can be accepted for publication. You may also include additional comments for the author, including concerns about research or publication ethics.

You may also provide optional suggestions and comments to authors that they might find helpful in planning their study.

Reviewer #1: 1. The authors are encouraged to provide a brief clarification regarding the novelty of the present systematic review compared with previously published methodological reviews on concentration-QT/QTc modelling. This would help readers better understand the specific knowledge gap that this review intends to address.

2. The eligibility criteria are generally well described; however, the authors may consider adding a few practical examples of eligible and non-eligible studies, particularly regarding studies using Cmax/AUC-based exposure metrics versus direct concentration-QT/QTc models, to improve transparency during study selection.

3. The authors may consider clarifying how disagreements between reviewers will be managed during data extraction when multiple eligible models or multiple QT correction methods are reported within the same study.

4. The manuscript describes several possible model structures (linear, nonlinear, effect-compartment, and joint parent-metabolite models). The authors may consider providing additional details on how model-specific parameters will be compared or summarized when direct quantitative pooling is not feasible.

5. The concentration-QT/QTc-specific methodological appraisal framework is a useful component of this protocol. The authors may clarify whether this framework has been previously applied or whether it was specifically developed for this review, and how its reliability between reviewers will be assessed.

6. The authors have appropriately planned sensitivity analyses; however, they may consider explicitly defining which analyses will be considered exploratory in the final review to facilitate appropriate interpretation of the findings.

**********

what does this mean?). If published, this will include your full peer review and any attached files.

If you choose “no”, your identity will remain anonymous but your review may still be made public.

**Do you want your identity to be public for this peer review?**  For information about this choice, including consent withdrawal, please see our Privacy Policy

Reviewer #1: **Yes:**  Mahdi Ahmadinia

**********

[NOTE: If reviewer comments were submitted as an attachment file, they will be attached to this email and accessible via the submission site. Please log into your account, locate the manuscript record, and check for the action link "View Attachments". If this link does not appear, there are no attachment files.]

To ensure your figures meet our technical requirements, please review our figure guidelines: https://journals.plos.org/plosone/s/figures

You may also use PLOS’s free figure tool, NAAS, to help you prepare publication quality figures: https://journals.plos.org/plosone/s/figures#loc-tools-for-figure-preparation.

NAAS will assess whether your figures meet our technical requirements by comparing each figure against our figure specifications.

# Author comment: Author response to Decision Letter 1

**DOI:** 10.1371/journal.pone.0357131.r002

25 Sep 2026

Dear Dr. Nanzigu and Reviewer,

We sincerely thank the Academic Editor and Reviewer for their careful and constructive assessment of our manuscript. We appreciate the positive evaluation of the rationale, methodological rigor, reproducibility, data-sharing plan, and presentation of the protocol. We have revised the manuscript to address all comments in detail. The principal revisions include clarification of the novelty of the review relative to previous methodological publications, addition of operational examples for study eligibility, further specification of procedures for selecting among multiple eligible models and QT correction methods, expansion of the synthesis plan for heterogeneous model structures, clarification and reliability assessment of the review-specific concentration–QT/QTc methodological framework, and explicit identification of exploratory analyses. We have also addressed all journal technical requirements, including the Data Availability Statement and Figure 1.

All changes are highlighted in the marked-up manuscript. Our point-by-point responses are provided below.

PONE-D-26-40572

Clinical Pharmacokinetic-Pharmacodynamic Relationships between Drug Exposure and the QT/QTc Interval: Protocol for a Systematic Review and Quantitative Synthesis

PLOS One

Journal Requirements:

When submitting your revision, we need you to address these additional requirements.

1. Please ensure that your manuscript meets PLOS ONE's style requirements, including those for file naming. The PLOS ONE style templates can be found at

https://journals.plos.org/plosone/s/file?id=wjVg/PLOSOne_formatting_sample_main_body.pdf and

https://journals.plos.org/plosone/s/file?id=ba62/PLOSOne_formatting_sample_title_authors_affiliations.pdf

Response: Thank you. We have reviewed the revised manuscript and all accompanying files for compliance with the PLOS ONE formatting and file-naming requirements. The clean manuscript, marked-up manuscript, figure file, and Supporting Information files have been prepared accordingly.

2. When completing the data availability statement of the submission form, you indicated that you will make your data available on acceptance. We strongly recommend all authors decide on a data sharing plan before acceptance, as the process can be lengthy and hold up publication timelines. Please note that, though access restrictions are acceptable now, your entire data will need to be made freely accessible if your manuscript is accepted for publication. This policy applies to all data except where public deposition would breach compliance with the protocol approved by your research ethics board. If you are unable to adhere to our open data policy, please kindly revise your statement to explain your reasoning and we will seek the editor's input on an exemption. Please be assured that, once you have provided your new statement, the assessment of your exemption will not hold up the peer review process.

Response: Thank you for this clarification. We have revised the Data Availability Statement to accurately reflect the nature of the present article as a study protocol. No datasets have yet been generated or analysed for this protocol manuscript.

3. Please upload a new copy of Figure 1 as the detail is not clear. Please follow the link for more information: https://journals.plos.org/plosone/s/figures.

Response: Thank you. Figure 1 has been revised to improve clarity and readability. The text size and visual elements have been optimized, and a new high-resolution version of the figure has been uploaded in accordance with the PLOS ONE figure specifications. The scientific content of the figure has not changed.

4. If the reviewer comments include a recommendation to cite specific previously published works, please review and evaluate these publications to determine whether they are relevant and should be cited. There is no requirement to cite these works unless the editor has indicated otherwise.

Response: We have evaluated the literature relevant to the reviewer’s comments and have added relevant references where they directly support the revised manuscript. No reference was added solely because of a citation recommendation.

5. Please review your reference list to ensure that it is complete and correct. If you have cited papers that have been retracted, please include the rationale for doing so in the manuscript text, or remove these references and replace them with relevant current references. Any changes to the reference list should be mentioned in the rebuttal letter that accompanies your revised manuscript. If you need to cite a retracted article, indicate the article’s retracted status in the References list and also include a citation and full reference for the retraction notice.

Response: Thank you. We have carefully reviewed the entire reference list for completeness and bibliographic accuracy, including author names, article titles, journal information, publication year, pagination/article numbers, and DOI information. We also checked the cited literature for retraction status. Relevant corrections and newly added references are reflected in the revised manuscript.

Additional Editor Comments:

The proposal is well written with clear objectives and methods. However, there are a few comments from our reviewer which you need to address, and highlight the changes in your amended protocol.

Reviewer's Responses to Questions

Comments to the Author

1. Does the manuscript provide a valid rationale for the proposed study, with clearly identified and justified research questions?

The research question outlined is expected to address a valid academic problem or topic and contribute to the base of knowledge in the field.

Reviewer #1: Yes

________________________________________

2. Is the protocol technically sound and planned in a manner that will lead to a meaningful outcome and allow testing the stated hypotheses?

The manuscript should describe the methods in sufficient detail to prevent undisclosed flexibility in the experimental procedure or analysis pipeline, including sufficient outcome-neutral conditions (e.g. necessary controls, absence of floor or ceiling effects) to test the proposed hypotheses and a statistical power analysis where applicable. As there may be aspects of the methodology and analysis which can only be refined once the work is undertaken, authors should outline potential assumptions and explicitly describe what aspects of the proposed analyses, if any, are exploratory.

Reviewer #1: Yes

________________________________________

3. Is the methodology feasible and described in sufficient detail to allow the work to be replicable?

Descriptions of methods and materials in the protocol should be reported in sufficient detail for another researcher to reproduce all experiments and analyses. The protocol should describe the appropriate controls, sample size calculations, and replication needed to ensure that the data are robust and reproducible.

Reviewer #1: Yes

________________________________________

4. Have the authors described where all data underlying the findings will be made available when the study is complete?

The PLOS Data policy requires authors to make all data underlying the findings described in their manuscript fully available without restriction, with rare exception, at the time of publication. The data should be provided as part of the manuscript or its supporting information, or deposited to a public repository. For example, in addition to summary statistics, the data points behind means, medians and variance measures should be available. If there are restrictions on publicly sharing data—e.g. participant privacy or use of data from a third party—those must be specified.

Reviewer #1: Yes

________________________________________

5. Is the manuscript presented in an intelligible fashion and written in standard English?

PLOS ONE does not copyedit accepted manuscripts, so the language in submitted articles must be clear, correct, and unambiguous. Any typographical or grammatical errors should be corrected at revision, so please note any specific errors here.

Reviewer #1: Yes

________________________________________

6. Review Comments to the Author

Reviewer #1: 1. The authors are encouraged to provide a brief clarification regarding the novelty of the present systematic review compared with previously published methodological reviews on concentration-QT/QTc modelling. This would help readers better understand the specific knowledge gap that this review intends to address.

Response: Thank you for this helpful suggestion. We have expanded the Introduction to more explicitly distinguish the present systematic review from previous methodological reviews, regulatory perspectives, and practical tutorials on concentration–QT/QTc modelling.

2. The eligibility criteria are generally well described; however, the authors may consider adding a few practical examples of eligible and non-eligible studies, particularly regarding studies using Cmax/AUC-based exposure metrics versus direct concentration-QT/QTc models, to improve transparency during study selection.

Response: We agree and have added practical operational examples immediately following the eligibility criteria. We now explicitly distinguish direct time-matched concentration–QT/QTc analyses from studies using continuous summary exposure measures such as Cmax, Cmin, or AUC. We also clarify that analyses based solely on dose groups, categorical exposure strata, or simulation-only exposure profiles without original human concentration–QT/QTc data are not eligible. This addition is intended to improve reproducibility of study selection.

3. The authors may consider clarifying how disagreements between reviewers will be managed during data extraction when multiple eligible models or multiple QT correction methods are reported within the same study.

Response: We have clarified the adjudication procedure for studies reporting multiple eligible models or QT correction methods. Both reviewers will independently extract all eligible analyses and apply the prespecified hierarchy. Alternative models and correction methods will be retained rather than discarded, while the prespecified primary estimate will be selected for the principal synthesis. Any disagreement regarding model classification or selection will be resolved by consensus or third-reviewer adjudication and documented in the review decision log.

4. The manuscript describes several possible model structures (linear, nonlinear, effect-compartment, and joint parent-metabolite models). The authors may consider providing additional details on how model-specific parameters will be compared or summarized when direct quantitative pooling is not feasible.

Response: We agree that the handling of heterogeneous model structures required further specification. We have expanded the Data synthesis section to describe model-specific synthesis explicitly. Linear slopes, Emax/EC50 parameters, sigmoid Emax parameters, delayed-effect parameters, and parent–metabolite model estimates will be retained in their native parameterisation. Structurally different models will not be forced into a common linear effect measure. When quantitative pooling is not justified, estimates will be grouped by active moiety, estimand, and model class and summarised using structured tables and SWiM principles. Comparable model-predicted QT/QTc effects at clinically relevant exposures will also be considered as clinically interpretable summaries where appropriate.

5. The concentration-QT/QTc-specific methodological appraisal framework is a useful component of this protocol. The authors may clarify whether this framework has been previously applied or whether it was specifically developed for this review, and how its reliability between reviewers will be assessed.

Response: We have clarified that the concentration–QT/QTc-specific framework was developed specifically for this review and is not presented as a previously validated standalone risk-of-bias instrument. It supplements established design-based tools and was informed by ICH E14/S7B and concentration-QTc pharmacometric guidance. We have also added a prespecified calibration and reliability procedure. Two reviewers will independently pilot the framework on methodologically diverse studies before formal application, and initial domain-level agreement will be documented using percentage agreement and, where estimable, Cohen’s κ with 95%CI. Disagreements will subsequently be resolved by consensus or third-reviewer adjudication.

6. The authors have appropriately planned sensitivity analyses; however, they may consider explicitly defining which analyses will be considered exploratory in the final review to facilitate appropriate interpretation of the findings.

Response: We have added an explicit distinction between primary, supportive sensitivity, and exploratory analyses. Meta-regression, heterogeneity-driven subgroup analyses, molar-scale presentations, analyses of non-primary models, estimates requiring substantial reconstruction, and any unplanned analyses prompted by the observed evidence will be identified as exploratory. Any post hoc analysis will be labelled explicitly in the final review and will not be interpreted as confirmatory.

Attachment Submitted filename: Response to Reviewers.pdf

# Editor report: Decision Letter 1

**DOI:** 10.1371/journal.pone.0357131.r003

**Authors:** Sarah Nanzigu

30 Sep 2026

Clinical Pharmacokinetic-Pharmacodynamic Relationships between Drug Exposure and the QT/QTc Interval: Protocol for a Systematic Review and Quantitative Synthesis

PONE-D-26-40572R1

Dear Dr. Mohammadnezhad,

We’re pleased to inform you that your manuscript has been judged scientifically suitable for publication and will be formally accepted for publication once it meets all outstanding technical requirements.

Within one week, you’ll receive an e-mail detailing the required amendments. When these have been addressed, you’ll receive a formal acceptance letter and your manuscript will be scheduled for publication.

An invoice will be generated when your article is formally accepted. Please note, if your institution has a publishing partnership with PLOS and your article meets the relevant criteria, all or part of your publication costs will be covered. Please make sure your user information is up-to-date by logging into Editorial Manager at Editorial Manager® and clicking the ‘Update My Information' link at the top of the page. For questions related to billing, please contact billing support.

If your institution or institutions have a press office, please notify them about your upcoming paper to help maximize its impact. If they’ll be preparing press materials, please inform our press team as soon as possible -- no later than 48 hours after receiving the formal acceptance. Your manuscript will remain under strict press embargo until 2 pm Eastern Time on the date of publication. For more information, please contact onepress@plos.org.

Kind regards,

Sarah Nanzigu, Ph.D.,MSc.,MBchB

Academic Editor

PLOS One

Additional Editor Comments (optional):

Reviewers' comments:

# Editor report: Acceptance letter

**DOI:** 10.1371/journal.pone.0357131.r004

**Authors:** Sarah Nanzigu

PONE-D-26-40572R1

PLOS One

Dear Dr. Mohammadnezhad,

I'm pleased to inform you that your manuscript has been deemed suitable for publication in PLOS One. Congratulations! Your manuscript is now being handed over to our production team.

At this stage, our production department will prepare your paper for publication. This includes ensuring the following:

* All references, tables, and figures are properly cited

* All relevant supporting information is included in the manuscript submission,

* There are no issues that prevent the paper from being properly typeset

You will receive further instructions from the production team, including instructions on how to review your proof when it is ready. Please keep in mind that we are working through a large volume of accepted articles, so please give us a few days to review your paper and let you know the next and final steps.

Lastly, if your institution or institutions have a press office, please let them know about your upcoming paper now to help maximize its impact. If they'll be preparing press materials, please inform our press team within the next 48 hours. Your manuscript will remain under strict press embargo until 2 pm Eastern Time on the date of publication. For more information, please contact onepress@plos.org.

You will receive an invoice from PLOS for your publication fee after your manuscript has reached the completed accept phase. If you receive an email requesting payment before acceptance or for any other service, this may be a phishing scheme. Learn how to identify phishing emails and protect your accounts at https://explore.plos.org/phishing.

If we can help with anything else, please email us at customercare@plos.org.

Thank you for submitting your work to PLOS One and supporting open access.

Kind regards,

PLOS One Editorial Office Staff

on behalf of

Dr. Sarah Nanzigu

Academic Editor

PLOS One
